# Poor reporting quality of randomized controlled trials comparing treatments of COVID-19–A retrospective cross-sectional study on the first year of publications

**DOI:** 10.1371/journal.pone.0292860

**Published:** 2023-10-16

**Authors:** Linda Grüßer, Charlotte Eißing, Ana Kowark, András P. Keszei, Julia Wallqvist, Rolf Rossaint, Sebastian Ziemann

**Affiliations:** 1 Department of Anesthesiology, University Hospital RWTH Aachen, Aachen, Germany; 2 Department of Dermatology, Fachklinik Hornweide, Muenster, Germany; 3 Department of Anesthesiology and Intensive Care Medicine, University Hospital Bonn, Bonn, Germany; 4 Center for Translational & Clinical Research Aachen (CTC-A), Medical Faculty RWTH Aachen University, Aachen, Germany; CHU Nantes, FRANCE

## Abstract

**Introduction:**

Transparent and complete reporting of randomized controlled trials (RCTs) is essential for critical scientific appraisal of the results. It has been argued whether publications during the COVID-19 pandemic have met reporting standards. In this study, we assessed reporting adherence of RCTs on treatment interventions in COVID-19 patients to the CONSORT checklist and discuss which lessons can be learned to improve reporting in the future.

**Methods:**

This was a retrospective, cross-sectional study performed at the University Hospital RWTH Aachen, Germany. We conducted a pragmatic systematic literature search in the PubMed database to identify RCTs on treatment interventions in COVID-19 patients in the first year of publications on the topic (March 2020-February 2021). We investigated the adherence of each publication to the CONSORT checklist and assessed the association between specific predictors and percentage adherence in an exploratory multivariable regression model.

**Results:**

We analyzed 127 RCTs and found that the median percentage adherence to the CONSORT checklist was 54.3% [IQR 38.9 to 65.7]. In the exploratory multivariable regression model, the impact factor (highest tertile of impact factor compared to lowest tertile ß = 21.77, 95% CI 13.89 to 29.66, p<0.001; middle tertile compared lowest tertile ß = 11.79, 95% CI 5.74 to 17.84, p<0.001)) and authors’ referral to the CONSORT statement (ß = 9.29, 95% CI 2.98 to 15.60, p = 0.004) were associated with a higher percentage adherence to the CONSORT checklist.

**Conclusion:**

The reporting quality of RCTs on treatment interventions in COVID-19 patients during the first year of publications was poor. Measures to improve reporting quality are urgently needed.

## Introduction

Transparent and accurate reporting of clinical trials is essential to enable progress in medicine [1, 2]. Randomized controlled trials (RCTs), if properly designed and executed, are considered the gold standard for assessing healthcare interventions [1, 3]. However, biased results from inadequately designed, executed or reported trials can mislead clinical decision making [1, 4]. Inaccurate and incomplete reporting of RCTs has been found by various investigations leading to the development of the Consolidated Standards of Reporting Trial (CONSORT) statement [5, 6]. Published in 1996, this statement comprises evidence-based recommendations and a 25-item checklist that provide guidance to authors for transparent and complete reporting of RCTs [6]. The CONSORT checklist has since been revised twice and is endorsed by over 600 biomedical journals [1, 5–7].

In the first year of the COVID-19 pandemic, there was a tremendous demand for data on treatment of the new disease. Numbers of hospitalized patients and deaths were increasing worldwide, and clinicians and governments relied on timely publication of trials as the basis for clinical and public health decision making. Journals did not only face a surge of submitted publications on COVID-19, but were also under enormous pressure to expedite the review process [8]. Concerns regarding the quality of published research in this unprecedented situation have been expressed [9–12]. Numerous published studies have been retracted and queried [12]. A study comparing 686 COVID-19 articles to historical control publications demonstrated a shorter time to acceptance and lower methodological quality across all study designs [13]. The *reporting quality* of RCTs on treatment interventions in COVID-19 patients, which is pivotal for critical scientific appraisal of findings, has only been partially investigated. One study assessed the reporting quality on COVID-19 treatment interventions and found suboptimal adherence to the CONSORT guideline [14]. However, the authors identified only 53 RCTs until December 1, 2020 and applied a less rigorous evaluation regime [14]. The present study aimed to evaluate the reporting quality of RCTs within the complete first year after the first publication of a RCT on COVID-19. This time period presents one of the most challenging stressing tests in medical publishing history. It is hoped that a thorough and self-critical assessment of the scientific community’s performance in this field may lead to an improvement of reporting quality of RCTs in the future and hence support advancement in patient care.

## Methods

### Study design

We performed a retrospective cross-sectional study of scientific publications about RCTs investigating treatment interventions in COVID-19 patients that were published between March 1, 2020 and February 28, 2021. An ethical approval was not required. Our analysis is reported in concordance with the “STrengthening the Reporting of OBservational studies in Epidemiology” (STROBE) checklist [15]. The systematic literature search and analysis conducted for this study are reported adhering to the “Preferred Reporting Items for Systematic reviews and Meta-Analyses” (PRISMA) statement [16]. This retrospective observational study assessed characteristics of publications that were chosen based on a pragmatic yet systematic literature search. It was performed from a clinician’s perspective with the urgent need for information during the pandemic. Thus, it did not qualify for registration at clinical trial nor review database. A study protocol was not published. The decision to perform this study based on the lack of data in September 2020.

### Setting

This cross-sectional study was performed at the Department of Anesthesiology, University Hospital RWTH Aachen. The systematic literature search was conducted in the United States National Library of Medicine’s PubMed database. The latest search was performed on April 9, 2021.

### Selection of eligible studies

The first RCT meeting our inclusion criteria was published in March 2020. Hence, we searched publications that were published in the 12 months between March 2020 and February 2021. Our search based on the PubMed’s ‘clinical queries’–search filter which was developed by Haynes and colleagues and has been validated and used before [17–20]. Accordingly, we used the category ‘Therapy*’* optimized for ‘sensitivity/broad search’ to retrieve clinically relevant publications which focused on interventions to treat COVID-19 patients. Our search excluded the publication types case report, comment, editorial, letter, meta-analysis and review. The final search term was: (((Therapy/Broad[filter]) AND (COVID-19)) AND (("2020/03/01"[Date-Publication]: "2021/02/28"[Date-Publication]))) NOT ((((((Editorial[Publication Type]) OR (Case reports[Publication Type])) OR (Comment[Publication Type])) OR (Review[Publication Type])) OR (Meta-analysis[Publication Type])) OR (Letter[Publication Type])). One author (CE) screened all titles and abstracts for suitability of publication and topic type. Only original publications on randomized treatment interventions in COVID-19 patients were included. Publications not available in the English language, brief preliminary reports, or RCTs on other than treatment interventions in COVID-19 patients, e.g., vaccination, prevention therapy, post- or pre-exposure therapy were excluded. In case of uncertainty concerning inclusion of a study, two further authors (LG, SZ) were consulted, and a consentaneous decision was made. Due to the explorative nature of the study, no sample size calculation was performed.

### Data extraction

In order to standardize the evaluation of adherence to the CONSORT statement distinct requirements for every item of the CONSORT checklist were predefined in conformity with the CONSORT’s explanation and elaboration document [1], and based on the work of Stevanovic et al. [21]. The resulting evaluation data sheet contained 25 items of which 12 had subitems. Hence, the maximum achievable adherence was 37 points. All identified eligible publications (abstract and main text) were evaluated by assessing adherence to all 25 items and 12 subitems. As recommended by Turner et al. [22], a dichotomous rating scheme (“yes, requirement of item complete” or “no, requirement of item incomplete”) was used. Based on this rationale, an item was only rated complete when all required information was given. For 12 out of the 37 items, one or more checkpoints were implemented to ensure that all necessary requirements were assessed consistently and completely. E.g., item 4a ‘participants—eligibility criteria*’* was only rated “complete”, when information on the method of recruitment as well as on the inclusion and exclusion criteria were provided.

As we focused exclusively on the reporting quality of publications, study protocols were not investigated. Similar to Stevanovic et al. [21] we evaluated item 3b (‘method changes’), 6b (‘protocol deviations’), 7b (‘interim analysis and stopping guidelines’), 11a (‘blinding*’*), 12b (‘methods of additional analysis’), and 18 (‘results of additional analysis’) as “incomplete” if no information about the respective item was given. E.g., even if there were no protocol deviations, this needed to be reported.

Authors could reference to supplementary materials for extra information on an item. For items to be checked, the reference had to be specific (e.g., “more information on the eligibility criteria can be found in supplement”). However, following CONSORT recommendations, information on the randomization process (items 8–10) always had to be included in the body of the main article [1]. For the following two items, we added the option “not applicable”: 11b *(*‘similarity of interventions’) in case the trial was not blinded and 17b (‘reporting of absolute and relative effect size of binary outcomes*’*) in case no binary outcomes were used. After an initial analysis of 30 articles, the evaluation data sheet was reviewed by the three assessors (CE, LG, SZ) to detect potential misinterpretation and the wording of some items was adapted to improve objective comprehensibility. The final evaluation data sheet is presented in **S1 File.** Item adherence of all articles was evaluated in the full text (CE) and every uncertainty regarding evaluation of item adherence was solved in consensus by three authors (CE, LG, SZ).

### Bias

In order to minimize selection bias, PubMed’s clinical queries search strategy, which had been applied before, was used [18, 19]. In case the fulfillment of eligibility criteria was unclear, three authors were consulted and took a consentaneous decision. A random sample of 26 RCTs (20% of all included RCT) was cross-checked in order to validate an unambiguous application of the checklist (LG, SZ). Inter-rater reliability was evaluated using Cohen’s kappa. Similar to Stevanovic et al. and Ziemann et al., journals’ and authors’ names were not blinded due to the lack of evidence of this method to exclude bias [19, 21, 22]. Evaluation of item adherence was performed prior to analysis of impact factors.

### Outcomes

Primary outcome was the overall percentage of checklist items rated “complete” of all included publications. Secondary outcomes comprised the percentage of sufficiently rated checklist items for each item and sub-item, journal endorsement of the CONSORT checklist [6], authors’ referral to the CONSORT checklist or flowchart in their publication, and month of publication.

### Statistical analysis

Descriptive statistics are presented as mean (standard deviation) or median [interquartile range] for continuous variables, and number (percentage) for categorical variables. Each checklist item was weighted equally. Mann-Whitney-U test and Kruskal-Wallis test were used to investigate differences between two or multiple groups.

In an exploratory multiple linear regression analysis, we investigated the association between CONSORT checklist adherence and the potential predictors country of origin (defined as corresponding author’s affiliation), IF of journal (as given by Clarivate Analytics, Web of Science Group for 2021), month of publication, journal endorsement of the CONSORT checklist and author referral to the CONSORT checklist. These potential predictors of CONSORT-checklist adherence were identified based on prior knowledge. Categorical variables were transformed into dummy variables. First, univariable linear regression was performed for country of origin, IF, month of publication, journal endorsement and author referral to the CONSORT checklist or flowchart, and eventually an exploratory multivariable regression was fit to model the association between these potential predictors and percentage adherence to the CONSORT checklist. Graphical analysis via quantile-quantile (Q-Q) plot were used to assess normality. Assumptions of homoscedasticity and linearity were tested graphically. Coefficients, 95% Confidence Intervals (CI), standard error and p-values are reported. All analysis were conducted using STATA (BE Basic Edition 17.0, StataCorp LLC, Texas 77845, USA).

## Results

### Articles

**Fig 1:**
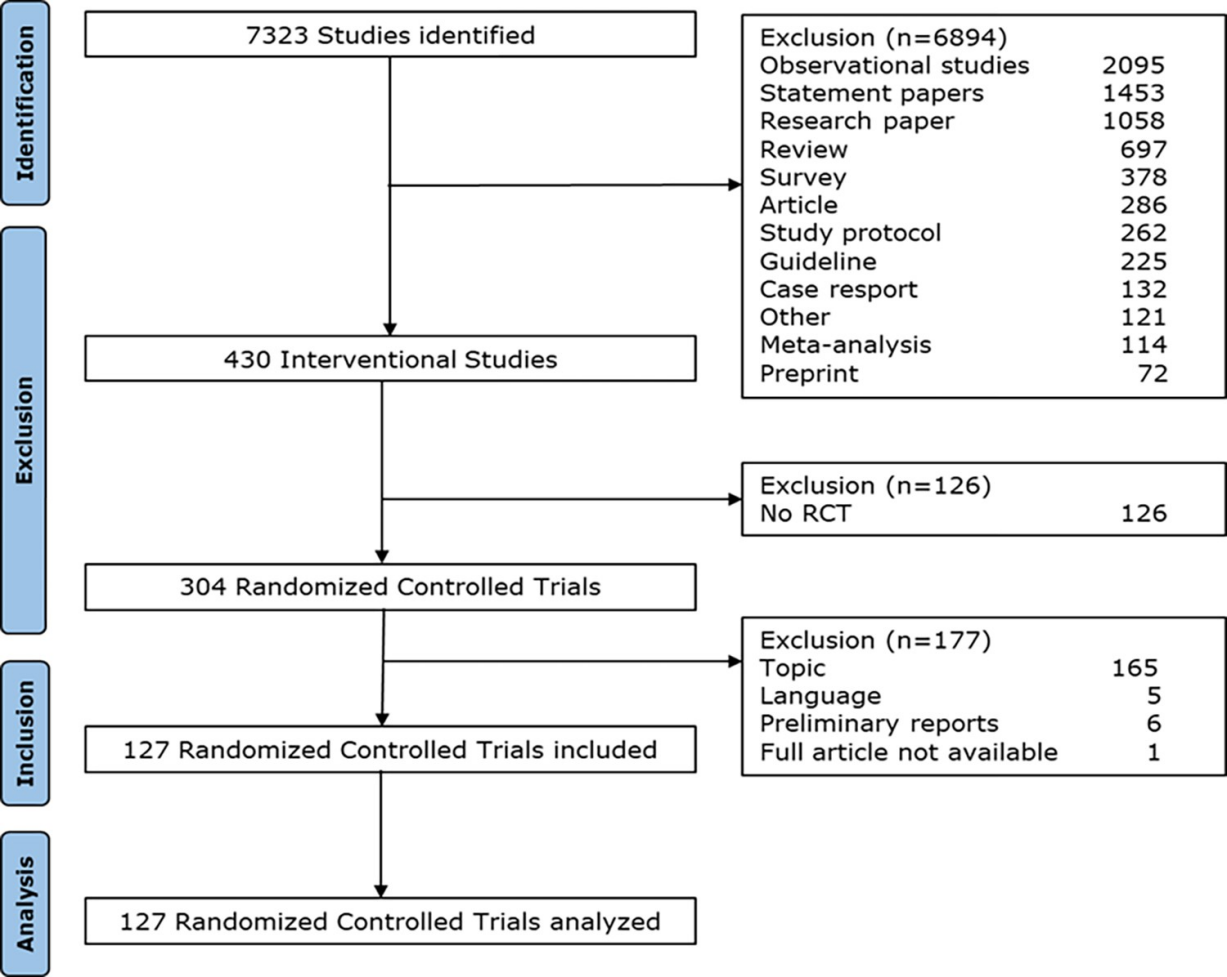
Flow chart.

Our search identified 7323 studies. After the manual screening of titles and abstract, a total of 430 interventional studies were identified, out of which 304 were RCTs. Out of these, 177 publications were excluded due to unsuitability of topic (e.g., unsuitable intervention or study population), language or being a brief preliminary report, see **Fig 1.** for details. Finally, 127 publications were included and analyzed upon adherence to the CONSORT checklist. A detailed list of all included publications is presented in **S2 File.**

### Characteristics of included studies

The majority of included RCTs reported the outcomes of pharmacological interventions (n = 116, 91.3%). Others covered e.g., oxygen therapy or prone positioning. Most of the investigated RCTs were two-armed (n = 108, 85.0%). More than half of the analyzed publications randomized at least 100 participants (n = 66, 52.0%). The smallest trial included 10 participants and the largest reported 7763 participants. Corresponding authors’ affiliations were located in 28 different countries. The majority of corresponding authors’ affiliations were in Asia (n = 60, 47.2%). Out of these, 29 publications were from China and 15 from Iran. Corresponding authors from institutions in North America made up for 22.8% (n = 29), in Europe 16.5% (n = 21) and in South America 11.0% (n = 14). Only three corresponding authors (2.4%) had their affiliation in Africa. In North America, corresponding authors’ affiliations were mostly located in the United States (n = 26), in Europe they were mostly located in Great Britain (n = 7), in South America mostly in Brazil (n = 12), and in Africa mostly in Egypt (n = 2). The 127 articles were published in 68 journals. The median impact factor was 9.1 [4.7 to 56.3], for 19 publications no impact factor was available yet. Out of the 127 investigated publications, 61 were published in journals that endorsed the CONSORT checklist. However, in only 31 publications of the 127 publications, authors referred to the checklist. The majority of publications was published in the second half of the observation period (n = 91, 71.7%).

### Main results

**Fig 2:**
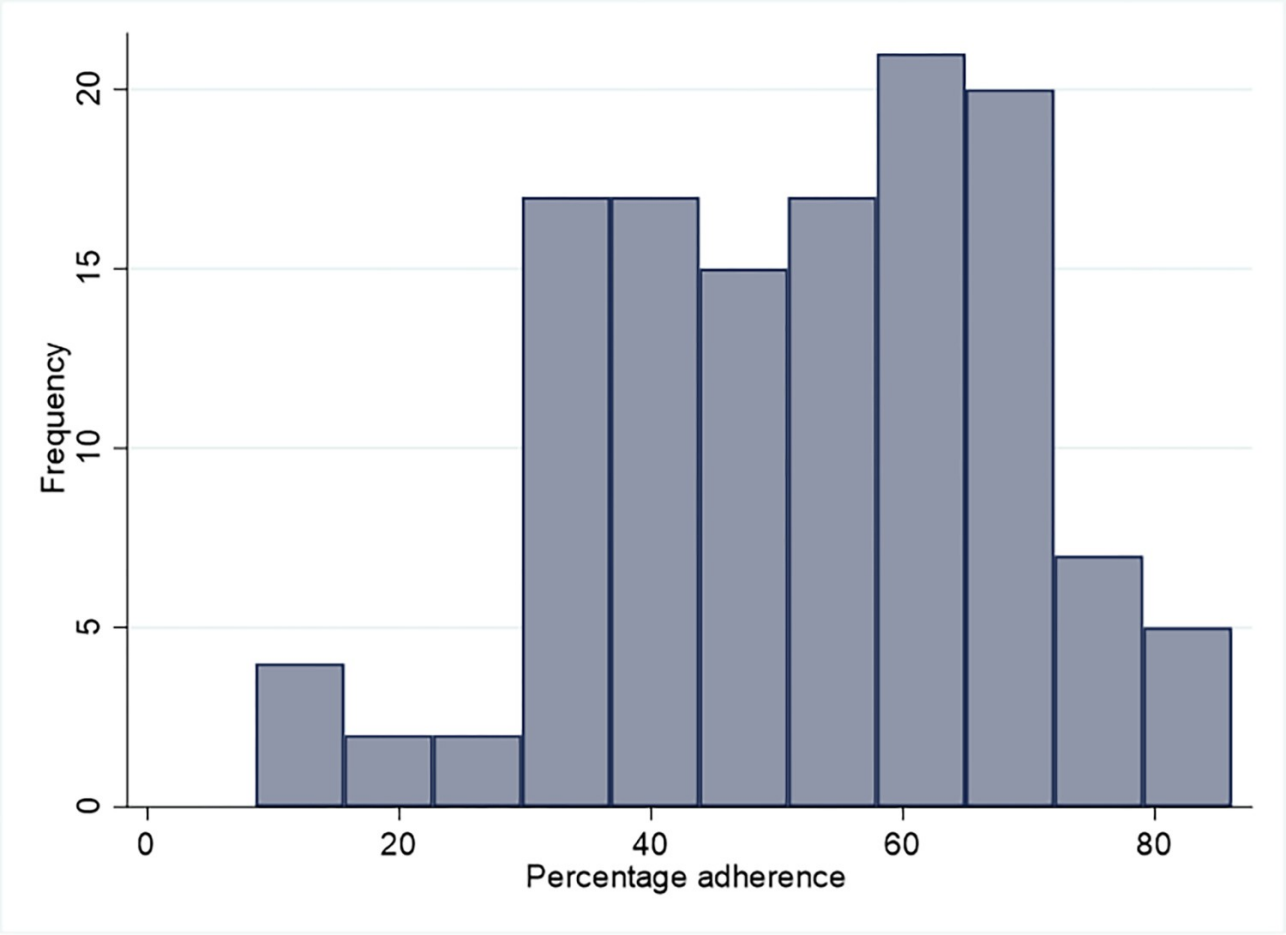
Frequency of the overall percentage adherence to the CONSORT checklist within the sample of 127 analyzed publications.

The overall mean percentage adherence to the CONSORT checklist was 52.4% (standard deviation:16.5) and the overall median percentage adherence was 54.3 [interquartile range: 38.9 to 65.7]. The individual percentage adherence of each investigated publication is shown in **S2 File**. The lowest percentage adherence was 8.6% and the highest was 86.1%. The frequency of the overall percentage adherence to the CONSORT checklist is presented in **Fig 2.**

The percentage adherence of all publications to each CONSORT checklist item is shown in **Table 1. Percentage adherence per item**. The most frequently sufficiently addressed items were *objectives and hypothesis* (n = 120, 94.5%) and presentation of ‘baseline demographic and clinical characteristics*’* (n = 119, 93.7%). The items least frequently sufficiently addressed were ‘implementation of the randomization process’ (n = 4, 3.1%) and ‘description of trial design’ (n = 8, 6.3%). Only 3.1% (n = 4) of the investigated publications adhered to all the recommendations regarding the reporting of information in the abstract, 76.4% (n = 97) used the word “randomized” in the title.

**Table 1:** Per item adherence.

Item	Part	Short description of item[1]	Number	Item adherence–detailed, n (%)	Item adherence–total, n (%)
1a	Title	“Randomized” in title	127	97 (76.4)	**97 (76.4)**
1b	Abstract	Contact information	127	124 (97.6)	**4 (3.1)**
		Trial design	127	18 (14.2)	
		Methods	127	5 (3.9)	
		Results	127	30 (23.6)	
		Conclusions	127	127 (100)	
		Registration	127	80 (63.0)	
		Funding	127	34 (26.8)	
		Headings	127	107 (84.3)	
		Concordance main article	127	120 (94.5)	
2a	Introduction	Rationale	127	126 (99.2)	**46 (36.2)**
		Scientific background: RCT	127	94 (74.0)	
		Scientific background: Systematic review	127	46 (36.2)	
2b	Introduction	Objectives and hypothesis	127	120 (94.5)	**120 (94.5)**
3a	Methods	Type of trial	127	33 (26.0)	**8 (6.3)**
		Allocation ratio	127	99 (78.0)	
		In case of drug trial: trial phase	122	20 (16.4)	
		In case of less common design: explanation	13	11 (84.6)	
3b	Methods	Method changes	127	24 (18.9)	**24 (18.9)**
4a	Methods	Method of recruitment	127	35 (27.6)	**35 (27.6)**
		Eligibility Criteria	127	124 (97.6)	
4b	Methods	Setting	127	36 (28.3)	**35 (27.6)**
		Location	127	115 (90.6)	
5	Methods	Intervention	127	114 (89.8)	**96 (75.6)**
		In case of drug trial: drug application	122	104 (85.3)	
		In case of drug trial: Drug name	122	122 (100)	
		In case of drug trial: Drug dosage	122	121 (99.2)	
		In case of drug trial: Timing	122	117 (95.9)	
6a	Methods	Definition of primary and secondary outcome measures	127	118 (92.9)	**107 (84.3)**
		Method of assessment	127	108 (85.0)	
6b	Methods	Protocol deviations	127	16 (12.6)	**16 (12.6)**
7a	Methods	Sample size	127	68 (53.5)	**68 (53.5)**
7b	Methods	Interim analysis and stopping guidelines	127	44 (34.7)	**44 (34.7)**
8a	Methods	Generation of random allocation sequence	127	63 (49.6)	**63 (49.6)**
8b	Methods	Type of randomization	127	78 (61.4)	**54 (42.5)**
		Restrictions	127	55 (43.3)	
9	Methods	Allocation concealment	127	52 (40.9)	**42 (40.9)**
10	Methods	Implementation—generation allocation sequence	127	33 (26.0)	**4 (3.1)**
		Implementation—patient enrollment	127	20 (15.8)	
		Implementation—assignment of interventions	127	23 (18.1)	
11a	Methods	Blinding	127	88 (69.3)	**88 (69.3)**
11b	Methods	If applicable: similarities of interventions	40	24 (60.0)	**24 (60.0)**
12a	Methods	Statistical methods for outcomes	127	114 (89.8)	**114 (89.8)**
12b	Methods	Methods for additional analyses	127	58 (45.7)	**57 (44.9)**
		Rationale for additional analyses	127	62 (48.8)	
13a	Results	Flow chart	127	72 (56.7)	**72 (56.7)**
13b	Results	Reasons for exclusion	127	111 (87.4)	**111 (87.4)**
14a	Results	Period of recruitment and follow up	127	87 (68.5)	**87 (68.5)**
14b	Results	Ending of trial	127	100 (78.7)	**100 (78.7)**
15	Results	Demographics (table)	127	124 (97.6)	**119 (93.7)**
		Continuous and asymmetrical variables	125	121 (96.8)	
		Categorical variables	124	119 (96.0)	
16	Results	Number of participants in each analysis	127	124 (97.6)	**103 (81.1)**
		Type of participants in each analysis	127	103 (81.1)	
17a	Results	Effect size and precision	127	59 (46.5)	**59 (46.5)**
17b	Results	In case of binary outcomes: absolute and relative effect size	67	9 (13.4)	**9 (13.4)**
18	Results	Additional analysis	127	46 (36.2)	**46 (36.2)**
19	Results	Adverse events	127	76 (59.8)	**76 (59.8)**
20	Discussion	Limitations	127	110 (86.6)	**27 (21.3)**
		Bias	127	27 (21.3)	
21	Discussion	Generalizability	127	90 (70.9)	**90 (70.9)**
22	Discussion	Interpretation in relation to other evidence	127	105 (82.7)	**105 (82.7)**
23	Further information	Registration number and name of trial registry	127	113 (89.0)	**113 (89.0)**
24	Further information	Reference to study protocol	127	66 (52.0)	**66 (52.0)**
25	Further information	Funding	127	113 (89.0)	**45 (35.4)**
		In case of funding: role of funders	113	56 (49.6)	
		In case of drug study: drug supply	124	66 (53.2)	
**Overall adherence to the CONSORT checklist**		mean (SD) median [IQR]	127	**52.4 (16.5)** **54.3 [38.9 to 65.7]**	

**Table 1**. This table shows the number and percentage adherence of the analyzed publications to each CONSORT item and the overall adherence to the CONSORT checklist. The short descriptions of the items refer to the original CONSORT checklist [1] and the data evaluation sheet of Stevanovic et al. [21].

### Further analysis

**Fig 3:**
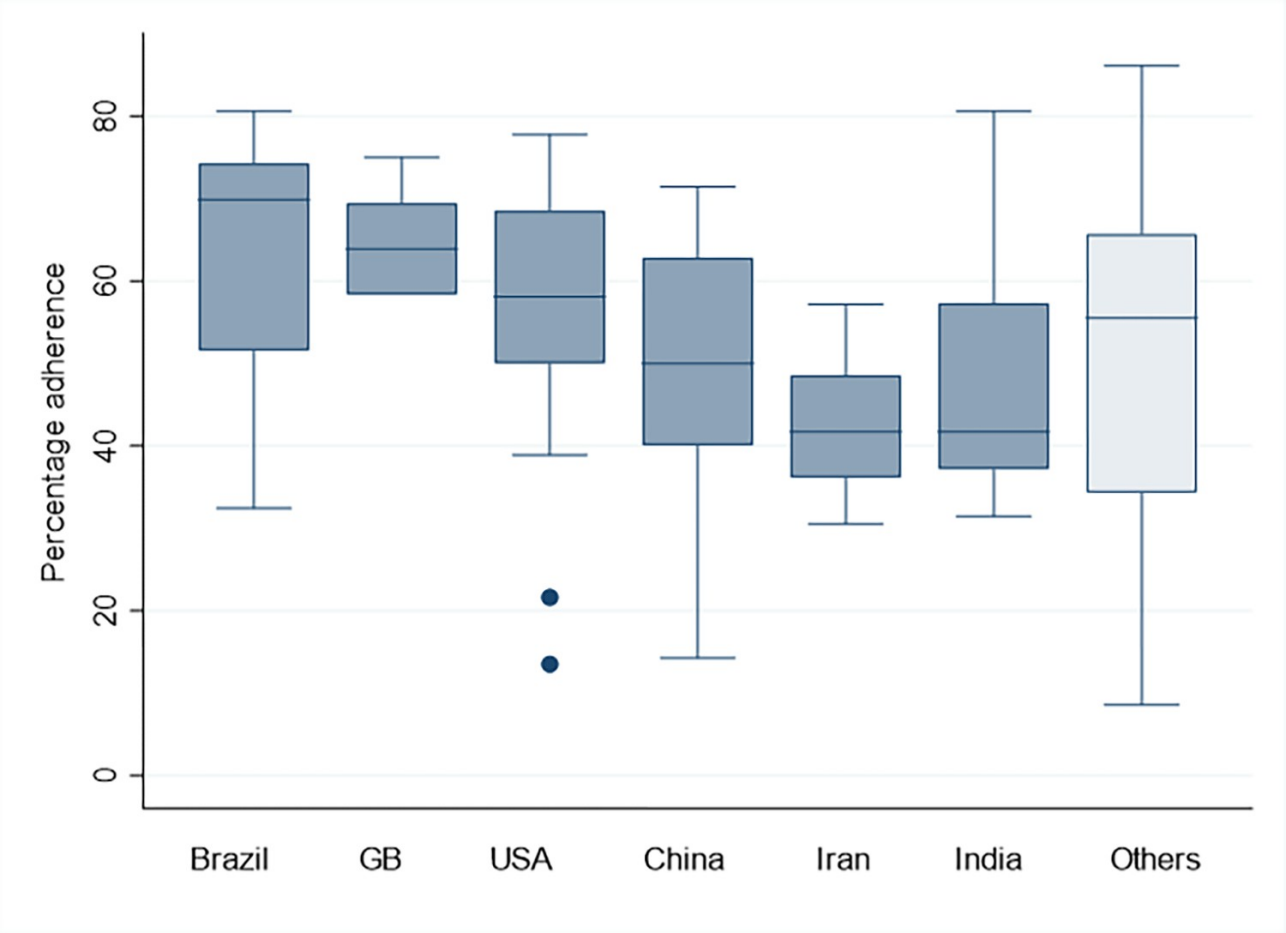
Percentage adherence to the CONSORT-checklist stratified by country of origin for the six countries with most publications.

We analyzed the 6 countries where most publications originated from, as determined by the affiliation of the corresponding author. Of these, from Brazil (n = 12) had a median percentage adherence of 69.9% [51.5 to 74.3], those from Great Britain (n = 7) had a median percentage adherence of 63.9 [58.3 to 69.4], followed by publications from USA (n = 26) with 58.1% [50 to 68.6], China (n = 29) with 50.0% [40.0 to 62.9], Iran (n = 15) with 41.7% [36.1 to 48.6] and India (n = 8) with 41.7% [37.1 to 57.3] **(Fig 3)** The Kruskal-Wallis test showed a difference in percentage adherence between the 6 different countries (χ2 = 22.9 with ties, p<0.001, with 5 d.f.).

No differences were found between publications published in different tertiles of month of publication (χ2 = 1.54 with ties, p = 0.46, with 2 d.f.). The Kruskal-Wallis test showed a difference in percentage adherence between different tertiles of impact factor (χ2 = 46.16 with ties; p<0.001, with 2 d.f.) We found a difference in distributions of percentage adherence of publications in journals that endorsed CONSORT (n = 61, percentage adherence 61.1 [45.7 to 69.4]) in comparison to journals that did not (n = 66, percentage adherence 46.6 [36.1 to 58.3]), z = -4.004, p<0.001. No difference was found in the median percentage adherence of publications in which authors referred to CONSORT (n = 31, percentage adherence 54.3% [41.7 to 67.6]) and publications in which authors did not (n = 96, percentage adherence 54.3% [37.1 to 63.9]), z = -0.618, p = 0.54) (**S3 File)**.

The coefficients of the linear regression models for each separate predictor are presented in **S4 File.** In the respective univariate models, journal endorsement of the CONSORT checklist was associated with an increase of 11.3 percentage points (95% CI: 5.8–16.8), p<0.001, whereas author referral to CONSORT was not associated with a difference in percentage adherence. Publications originating from Iran were associated with a decrease of -14.0 percentage points (95% CI: -24.0 to -3.9, p = 0.007) concerning percentage adherence in comparison to publications originating from the USA. The month published was neither associated with percentage adherence when modelled continuously nor when investigating tertiles. The impact factor was associated with a statistically significant increase of 0.25 percentage points (95% CI: 0.17 to 0.33, p<0.001) when modelled continuously and resulted in a difference of 15.2 percentage points (CI: 9.4 to 21.0, p<0.001) between publications published in the middle tertile in comparison to the lowest and a difference of 24.5 percentage points (95% CI 18.6 to 30.3, p<0.001) between publications published the highest tertile in comparison to the lowest.

In the exploratory multivariable model, the IF and author referral to CONSORT were shown to be predictors of percentage adherence to CONSORT (**Table 2**). As the assumption of linearity concerning the relationship between the IF and percentage adherence as well as between month of publication and percentage adherence could be questioned, we modelled these categorically by grouping them into tertiles. However, this did not change the overall directions of associations, nor the conclusions drawn from the model with IF and month of publication modelled as continuous variables **(S5 File)**. The variance inflation factor (VIF) did not detect multicollinearity (mean VIF = 1.74).

**Table 2:** Exploratory multivariate regression model for the percentage adherence to the CONSORT checklist.

Independent variable		Point estimate of change in percentage adherence			P-value
		β	95% CI	SE	
Journal endorsement CONSORT		2.17	-4.03 to 8.36	3.12	0.490
Author referral CONSORT		9.29	2.98 to 15.60	3.18	0.004
Country of origin^a^					
	Brazil	4.85	-4.28 to 13.99	4.60	0.294
	Great Britain	1.42	-9.01 to 11.86	5.26	0.787
	China	-4.25	-12.07 to 3.56	3.94	0.283
	India	-3.37	-14.79 to 8.05	5.75	0.559
	Iran	-7.63	-17.24 to 1.98	4.84	0.118
	Other countries	-0.24	-7.80 to 7.32	3.81	0.950
Impact factor					
	2^nd^ tertile	11.79	5.74 to 17.84	3.05	<0.001
	3^rd^ tertile	21.77	13.89 to 29.66	3.97	<0.001
Month of publication					
	2^nd^ tertile	-6.29	-12.55 to -0.03	3.15	0.049
	3^rd^ tertile	1.90	-4.43 to 8.24	3.19	0.552
Intercept		42.66	34.02 to 51.31	4.35	<0.001

^**a**^ Reference country: USA

The table shows the associations of the prespecified independent variables (predictors) with the percentage adherence to the CONSORT checklist according to a multiple linear regression model. Overall regression model: Number of observation n = 108 (19 publications published in journals without an available IF were excluded); R2 = 0.5155; adjusted R2 = 0.4543; F (12,97) = 8.42, p<0.001.

Interrater reliability of cross-checked items showed a kappa of 0.914.

## Discussion

In our retrospective cross-sectional study assessing the quality of publications on 127 RCT for the treatment of COVID-19, we found that the mean adherence to the CONSORT checklist was only 52.4%. In our exploratory multivariable regression model, the IF and authors’ referral to CONSORT were associated with a higher CONSORT adherence, whereas the month of publication or the respective journal’s endorsement were not.

The COVID-19 pandemic shed the spotlight on the scientific community, especially within the first year after the breakout of SARS-CoV-2. In face of rapidly increasing numbers of cases and deaths around the world results of RCTs were urgently needed—not only in order to guide clinical decision-making but also in order to inform further public actions. It has been questioned whether the scientific community stood the test of delivering high quality publications in such an unprecedented situation. There is now growing evidence that many publications did not meet the reporting standards scientific journals and researchers agreed upon in the CONOSRT statement [1, 14, 23, 24]. It has been argued that the novelty of the COVID-19 pandemic may have allowed for pragmatism around the study design, but that there is no reason to sacrifice transparent reporting [24].

The overall average percentage adherence of 52.4% found in our investigated publications calls for improvement. Scientists and clinicians already perform clinical studies according to ethical guidelines. It should be discussed whether maintaining transparent reporting standards constitutes an ethical imperative, too [24]–in particular in face of a global health threat with entire populations at risk, when trial findings are not only indispensable at the patient care level but also affect societies as a whole. Yin Y et al. investigated the reporting quality of RCTs on Covid-19 patients based on the CONSORT statement and found an average reporting rate of 53.9% [14]. However, they included only 53 publications and used a less detailed evaluation sheet [14]. In general, better, but admittedly still poor adherence rates have been found in top-ranked journals of critical care (61.3%) and anesthesiology (60%) prior to the pandemic [21, 25]. The median percentage adherence of publications published in journals in the highest tertile of IF in our study was 64.9 [58.3 to 71.4]. A before and after COVID-19 comparison is not appropriate as our search was not restricted to anesthesiology and intensive care related journals.

Several lessons can be learned from our assessment of reporting quality during the stressing test of the first year of COVID publications. First, only 76.4% of publications were identified as RCTs through their title and only 3.1% of publications followed *all* reporting recommendations in the abstract correctly. The mean percentage adherence concerning reported items in the abstract was 56.4%. When time and resources are scarce, the title and abstract may influence whether researchers and clinicians continue reading and hence can consider the findings in their own work. In some settings, the abstract remains the only information on a trial that is available underlining the importance of precise reporting [26]. In a different publication, Yin Y. et al. also investigated the quality of abstracts of publications on their identified 53 RCTs on patients with COVID-19 with the help of the elaborate CONSORT statement of abstracts [26] and found an average reporting rate of all abstract items of 50.2% [23]. Of note, less than half of the publications we investigated were assessed which could explain the slightly better yet still insufficient reporting quality of the publications analyzed in our work. The authors concluded that the poor reporting quality of abstracts could mitigate their usefulness and may mislead clinical decision making [23]. In addition, they highlight the importance of titles and abstracts for the conduction of systematic reviews and meta-analysis [23].

Concerning the main article, the CONSORT recommendations addressing the ‘trial design’ and ‘implementation of the randomization’ have hardly been followed sufficiently. Both items are imperative to evaluate the reliability and validity of findings. Authors should explain who implemented the three steps of a randomization process (consisting of “*generation of allocation sequence*, *process of allocation concealment*, *implementation of allocation concealment*”) so that readers can appraise the extend of bias [1, 27]. Encouragingly, the ‘objectives and hypothesis’ have been reported sufficiently in the majority of publications. This is in line with previous studies investigating the reporting quality of RCTs in the medical field [1, 21, 28]. In times of a unprecedented global pandemic, reporting about ‘baseline demographic and clinical characteristics*’* is crucial for readers to evaluate whether the presented findings are generalizable to their own population of interest. Authors of the broad majority of investigated publications followed this recommendation. Regarding the presentation of results, we found a low adherence of reporting the absolute and relative effect size of outcomes (item 17 b ‘In case of binary outcomes: absolute and relative effect size’). Reporting both is essential to understand and communicate the effect of an intervention and its implications to different audiences [1].

Typically, CONSORT endorsing journals include a mission statement in their instructions for authors, [6] and authors refer to it in their publications. Our results show that this is associated with a higher percentage adherence, which may demonstrate the success of these measures and suggest promising results of further actions in that direction. E.g., it would be desirable that even more journals endorse the CONSORT statement and that it becomes an integral part of a clinical researcher’s education. Publications of corresponding authors with affiliations in specific countries had higher adherence to the CONSORT statement than in others which could hint at the potential of educational efforts on a national level. Notably, almost twice as many journals endorsed CONSORT as authors referred to it. After adjusting for other variables, author referral to CONSORT remained an important predictor of percentage adherence whereas the association for journal endorsement was not as evident anymore. It could be argued that journals that endorse CONSORT should control more closely if authors are following the guidelines. However, the number of publications was limited to the first year of publications and we acknowledge that a higher number of observations could have yielded more precise estimations of associated variables. In general, it should be emphasized that the multiple regression analysis is purely explorative and prone to residual confounding. Another limitation is that we cannot exclude selection bias. First, we could have unintentionally excluded poorly reported studies that we did not identify as RCTs. Second, we only included publications written in English. Third, we used a systematic, yet pragmatic approach to search only one literature database. PubMed is one of the largest biomedical databases and widely used by clinicians accessing the medical literature. Even though our literature search may thus mimic clinical reality, we might have missed publications which were published in journals not indexed in PubMed. Further, we did not perform the entire search in duplication, which could have resulted in additional missed publications. Importantly, in ambiguous cases, three authors took a collective decision. The high inter-rater reliability of 0.914 suggests that the developed evaluation sheet is clearly structured, and definitions are objectively phrased. Of note, we limited our assessment to the CONSORT statement on parallel group randomized trials [1]. Additional CONSORT extensions are available for more specific trial designs [6].

Another limitation is that we only assessed information given in the main article, as this is what most readers limit their time to. Additional information could have been identified in appendices or in direct contact with authors. Interestingly, there is evidence that authors often used methodological safeguards in RCTs even though they did not report about it in their publication [29]. However, in order to make an adequate assessment of a RCT, all relevant information should be given in the main text. There is no justification to compromise internal validity of RCTs for speed or due to space. Our findings highlight that future authors and journal editors should pay special attention to commonly less frequently reported key items such as the completeness of information presented in the abstract, the implementation of the randomization process or the reporting of binary outcomes.

While a pandemic affects the entire global population, our search revealed that corresponding authors’ affiliations were not evenly distributed around the globe. This might be attributed to the fact that the pandemic affected countries at different times and speed. It might also reflect disparities in circumstances for conducting clinical trials and demonstrate that there is room for improvement concerning research collaborations with all countries. Noteworthy, we always chose the corresponding author’s affiliation’s country of origin. It is unknown, if reporting quality in international RCTs is different from single country studies.

It is hoped that our findings raise awareness of the importance of adequate reporting not only in face of a pandemic. Future studies are recommended to assess which measures prove to be most effective to improve reporting quality and evaluate progress over time.

The authors of CONSORT explain clearly that inadequate reporting and design are associated with biased estimates of treatment effects [1]. The relationship between reporting quality and clinical impact of a publication should be investigated to understand the importance of adequate reporting in more detail.

The CONSORT statement has been developed by experts from the field of clinical trial methodology, guideline development, knowledge translation and medical publication, and is endorsed by authors and editors [6]—there is no reason not to use it. Rather, scientific work can only be appreciated by the scientific community, patients and society when reported transparently.

## Conclusion

In order to advance evidence-based patient care and guide public health decisions it is important that readers can appraise the reliability and validity of research findings. Our results show that the reporting quality of RCTs according to the CONSORT statement in the first year of publications was poor. In our exploratory regression model, the impact factor was associated with a higher percentage adherence. We found that authors’ referral of the CONSORT statement may leverage reporting quality of RCTs, but further measures are urgently needed.

## Supporting information

S1 File Evaluation data sheet. (PDF)

S2 File Investigated publications. (PDF)

S3 File Boxplots of investigated variables. (PDF)

S4 File Linear regression models for separate predictors. (PDF)

S5 File Exploratory multiple regression model with continuous variables. (PDF)
