# Correction: Poor reporting quality of randomized controlled trials comparing treatments of COVID-19–A retrospective cross-sectional study on the first year of publications

**DOI:** 10.1371/journal.pone.0343360

**Published:** 2026-02-19

**Authors:** Linda Grüßer, Charlotte Eißing, Ana Kowark, András P. Keszei, Julia Wallqvist, Rolf Rossaint, Sebastian Ziemann

There are errors in the Author Contributions. The correct contributions are:

Conceptualization: Sebastian Ziemann, Ana Kowark

Data curation: Charlotte Eißing, Sebastian Ziemann, Linda Grüßer

Formal analysis: Linda Grüßer, András P. Keszei

Investigation: Charlotte Eißing, Linda Grüßer, Sebastian Ziemann

Methodology: Linda Grüßer, Sebastian Ziemann, Ana Kowark, András P. Keszei,

Charlotte Eißing

Project administration: Sebastian Ziemann, Rolf Rossaint

Resources: Rolf Rossaint

Supervision: Sebastian Ziemann, Rolf Rossaint

Validation: Ana Kowark, Sebastian Ziemann, Julia Wallqvist, Rolf Rossaint

Visualization: Linda Grüßer, Charlotte Eißing

Writing – original draft: Linda Grüßer

Writing – review & editing: Sebastian Ziemann, Julia Wallqvist, Charlotte Eißing, András P. Keszei, Rolf Rossaint, Ana Kowark

There are errors in the author affiliations. The correct affiliations are as follows:

Linda Grüßer^1^, Charlotte Eißing^1,2^, Ana Kowark^3^, Andra´s P. Keszei^4^, Julia Wallqvist^1^, Rolf Rossaint^1^, Sebastian Ziemann^1^

**1** Department of Anesthesiology, University Hospital RWTH Aachen, Aachen, Germany, **2** Department of Dermatology, Fachklinik Hornheide, Muenster, Germany, **3** Department of Anesthesiology and Intensive Care Medicine, University Hospital Bonn, Bonn, Germany, **4** Center for Translational & Clinical Research Aachen (CTC-A), Medical Faculty RWTH Aachen University, Aachen, Germany

## References

[1] Grüßer L, Eißing C, Kowark A, Keszei AP, Wallqvist J, Rossaint R, et al. Poor reporting quality of randomized controlled trials comparing treatments of COVID-19–A retrospective cross-sectional study on the first year of publications. PLoS One. 2023;18(10):e0292860. doi: 10.1371/journal.pone.0292860 37844082 PMC10578566

