# Supplementary material for: Poor reporting quality of randomized controlled trials comparing treatments of COVID-19–A retrospective cross-sectional study on the first year of publications
Source: PLoS One. 2023 Oct 16;18(10):e0292860. doi: 10.1371/journal.pone.0292860 (PMC10578566; doi:10.1371/journal.pone.0292860)
Supplement: S1 File — (PDF) [file pone.0292860.s001.pdf]

CONSORT checklist of information to include when reporting a randomized trial

Adapted from Moher et al.[1] and Stevanovic et al.[2]

Section/Topic	Item No	Description	Item present?	Yes	No	N.a.
**Title and abstract**						
	1a	"Randomized" trial is explicitly stated **in the title**.	☐	☐	☐	
	1b	The abstract provides a short, structured summary of what was **done and found.**		☐	☐	
		- *Short summary of the main article, including*				
		> *Contact details of the corresponding authors*	☐			
		> *Trial design (e.g. parallel, cluster, non-inferiority)*	☐			
		> *Methods (Eligibility criteria, Interventions, Objective, Outcome, Allocation generation and concealment, Blinding)*	☐			
		> *Results (Numbers randomized, recruitment status, numbers analyzed, primary outcome incl. effect size, harms)*	☐			
		> *Conclusions*	☐			
		> *Trial registration*	☐			
		> *Funding*	☐			
		- *The authors made use of a series of headings*	☐			
		- *Content abstract*: only information is given that is also provided in the main article	☐			
**Introduction**						
	2a	Explanation of		☐	☐	
		- Rationale (including justification for new trial and topic)	☐			
		- Scientific background, including at least evidence from				
		> a relevant trial (or absence of such)	☐			
		> a relevant systemic review	☐			
	2b	Specific **objectives or hypotheses** are stated in the introduction.	☐	☐	☐	
**Methods**						
**Trial design**						
	3a	Description of the trial design in methods is given, including		☐	☐	
		- Type of trial (e.g. parallel, cross-over, cluster, multi-arm including number of groups)	☐			
		– Allocation ratio is given	☐			
		– In case of drug trials the phase of the trial (I-IV) is described.	☐			
		– In case of less common design (e.g. adaptive platform trial), explanation of choice.	☐			
	3b	Any method changes after trial commencement are clearly described. If there are none, this is also reported.	☐	☐	☐	
**Participants**						
	4a	Participant **eligibility criteria** are stated clearly.		☐	☐	
		– Method of recruitment	☐			

		− Inclusion- and exclusion criteria	☐			
	4b	Clear statement of<br>**- Setting** (number and type of participating centre(s))<br>**- Location** (country, if applicable also city)	 ☐<br>☐	☐	☐	
**Interventions**						
	5	- Exact description of the **intervention** for the study- / control-group.<br><br>When drugs were used, description of:<br>− Drug application<br>− Name of the drug<br>− Dosage<br>- Time	☐<br><br><br>☐<br>☐<br>☐<br>☐	☐	☐	
**Outcome**						
	6a	Exact definition of **outcome**:<br>− Primary and secondary outcome measures defined<br>− Method of assessment	 ☐<br>☐	☐	☐	
	6b	**Deviations from the trial protocol**, regarding the outcome measures, are reported (including reason for deviation). In case there are no deviations, this is also reported.	☐	☐	☐	
**Sample Size**						
	7a	Detailed **sample size calculation** was performed. Explanation is given when sample size differed from originally intended size.	☐	☐	☐	
	7b	**Interim analyses** and **stopping guidelines** are reported in the methods part. If there were none, this is also reported.	☐	☐	☐	
**Random sequence generation**						
	8a	Method used for the generation of the **random allocation sequence** is clearly stated in **Methods** part.<br>− Detailed information of the process of randomization is given in <u>main article</u>, not in supplementary file	☐	☐	☐	
	8b	The <u>Method part</u> should include a detailed description of<br>− Type of **randomization** (e.g. simple randomization, block randomization, stratified randomization)<br>− Any **restriction** (e.g. stratification factors, block size, absence of restriction). In case of no restriction, stating “simple randomization” is sufficient.	 ☐<br>☐	☐	☐	
**Allocation concealment**						
	9	Detailed description of **methods** used to ensure **allocation concealment**.	☐	☐	☐	
**Implementation**						
	10	All **3 steps of randomizing** participants into the trial are described, including<br>− who generated the allocation sequence<br>− who was involved in the enrolment of participants<br>− who assigned participants to interventions	 ☐<br>☐<br>☐	☐	☐	
**Blinding**						

	11a	Detailed **description of blinding**, including who was blinded and how. It is not sufficient to simply mention “double-blind” as description.<br>- In case the trial was not blinded there must be reporting of such (e.g. as “blinding was not performed”)	☐<br>☐	☐	☐	
	11b	Description of **similarities of the interventions** (e.g. appearance, method of administration), to assure blinding of participants/healthcare providers) are reported<br><br>NA: Similarity of interventions not necessary/relevant for this trial, as it was not blinded.	☐	☐	☐	☐
**Statistical methods**						
	12a	Every **statistical method** used for the analysis of the primary and secondary outcomes should be mentioned. Only one dataset per participant is evaluated.	☐	☐	☐	
	12b	Description of the methods and rationale for any **additional analyses** is stated, e.g. subgroup analysis of adjusted analyses. In case there were none, this is stated<br>– Methods<br>– Rationale	 <br>☐<br>☐	☐	☐	

**Results**						
**Participant flow**						
	13a	Correct presentation of the study in the **participant flow**. (Number of participants randomized, received the intended treatment and analysed for primary outcome per group)	☐	☐	☐	
	13b	For each group, all **reasons of exclusions** after randomization are stated.	☐	☐	☐	
**Recruitment**						
	14a	The period of recruitment and follow up is clearly stated with dates in **methods or results** part.	☐	☐	☐	
	14b	It is clearly described why the trial ended, either in the methods or results part of the paper.	☐	☐	☐	
**Baseline data**						
	15	A <u>table</u> showing the baseline **demographical and clinical characteristics** of each group is present.<br>– Characteristics<br>– Continuous variables are given as mean with standard deviation (SD), asymmetrically distributed variables are given as median and interquartile range (IQR)<br>– Categorical values are given as numbers and proportions	 ☐<br>☐<br><br>☐	☐	☐	
**Number analyzed**						
	16	– The **number of participants**, included in each analysis, is reported.<br>– It is clearly described which participants were included in the **data-analysis**	☐<br>☐	☐	☐	
**Outcome & estimation**						
	17a	The estimated **effect size and its precision** (such as confidence intervals) should be reported for both primary and secondary outcomes for each group.	☐	☐	☐	
	17b	In case the **primary outcome is binary**, both the absolute and relative effect size are reported.<br>NA: No binary outcomes are used.	☐	☐	☐	☐
**Ancillary analyses**						

	18	The results from any **additional analyses** undertaken have to reported. It has to be mentioned if these were **pre-specified or subsequent analyses** and why they were performed. If **no additional analyses** were performed, this has to be stated as well.	☐	☐	☐	
Harms						
	19	The frequency and absolute risk of each adverse event (including the absence of it) per group are reported.	☐	☐	☐	

**Discussion**						
**Limitations**						
	20	All study **limitations and potential bias** (also absent) are clearly reported.<br>- Limitations (e.g. imprecision)<br>- Bias	 ☐<br>☐	☐	☐	
**Generalizability**						
	21	It is stated, if the findings can be generalized to other circumstances.<br>In case the results can <u>only be applicable to their study</u> circumstances and that further trials are needed to proof their results, this has to be stated.	☐	☐	☐	
**Interpretation**						
	22	Interpretation of the results of the present trial and a brief review of the **present trial's results in relation to other relevant evidence** (either from a relevant trial or systematic review) is given, either in Results or Discussion part. If there are no other studies, this is stated.	☐	☐	☐	

**Other information**						
**Registration**						
	23	**Registration number and name** of trial registry are provided, or reasons for not performed registration are explicitly stated.	☐	☐	☐	
**Protocol**						
	24	It is explicitly reported, where the **complete trial protocol** can be accessed.	☐	☐	☐	
**Funding**						
	25	**Financial funding and support** are clearly reported.<br>− In case of funding: role of funders is reported<br>− In case of drug studies, the supply of the drug is specified.	☐<br>☐<br>☐	☐	☐	

<u>**References**</u>:

1. Moher D, Hopewell S, et al. CONSORT 2010 explanation and elaboration: updated guidelines for reporting parallel group randomised trials. Int J Surg 2012; **10**:28-55.
2. Stevanovic A, Schmitz S, et al. CONSORT item reporting quality in the top ten ranked journals of critical care medicine in 2011: a retrospective analysis. PLoS One 2015; **10**:e0128061.
