# Supplementary material for: Poor reporting quality of randomized controlled trials comparing treatments of COVID-19–A retrospective cross-sectional study on the first year of publications
Source: PLoS One. 2023 Oct 16;18(10):e0292860. doi: 10.1371/journal.pone.0292860 (PMC10578566; doi:10.1371/journal.pone.0292860)
Supplement: S2 File — (PDF) [file pone.0292860.s002.pdf]

Supplementary S2. Investigated publications

PMID	Title	Author	Journal	DOI	Impact Factor 2021	Country of Origin*	Number of participants	Topic	Study arms	Percentage adherence
**32187464**	A Trial of Lopinavir–Ritonavir in Adults Hospitalized with Severe Covid-19	Cao B, et al.	NEJM	10.1056/NEJMoa2001282	91.25	China	199	Pharmacological intervention	two-armed	62.86
**32330277**	Effect of High vs Low Doses of Chloroquine Diphosphate as Adjunctive Therapy for Patients Hospitalized With Severe Acute Respiratory Syndrome Coronavirus 2 (SARS-CoV-2) Infection: A Randomized Clinical Trial	Borba MGS, et al.	JAMA network open	10.1001/jamanetworkopen.2020.8857	8.48	Brazil	81	Pharmacological intervention	two-armed	70.27
**32401715**	Triple combination of interferon beta-1b, lopinavir-ritonavir, and ribavirin in the treatment of patients admitted to hospital with COVID-19: an open-label, randomised, phase 2 trial	Hung IF, et al.	The Lancet	10.1016/S0140-6736(20)31042-4	79.32	China	86	Pharmacological intervention	two-armed	68.57
**32409561**	Hydroxychloroquine in patients with mainly mild to moderate coronavirus disease 2019: open label, randomised controlled trial	Tang W, et al.	BMJ	10.1136/bmj.m1849	39.89	China	150	Pharmacological intervention	two-armed	69.44
**32423584**	Remdesivir in adults with severe COVID-19: a randomised, double-blind, placebo-controlled, multicentre trial	Wang Y, et al.	The Lancet	10.1016/S0140-6736(20)31022-9	79.32	China	237	Pharmacological intervention	two-armed	63.89
**34775361**	Efficacy and safety of Lianhuaqingwen	Hu K, et al.	Phytomedicine	10.1016/j.phy	5.34	China	284	Traditional Medicine	two-armed	41.67

	capsules, a repurposed Chinese herb, in patients with coronavirus disease 2019: A multicenter, prospective, randomized controlled trial			med.2021.153800						
**32445440**	Remdesivir for the Treatment of Covid-19 - Final Report	Beigel JH, et al.	NEJM	10.1056/NEJMoa2007764	91.25	USA	1062	Pharmacological intervention	two-armed	61.11
**32459919**	Remdesivir for 5 or 10 Days in Patients with Severe Covid-19	Goldman JD, et al.	NEJM	10.1056/NEJMoa2015301	91.25	USA	397	Pharmacological intervention	two-armed	60.00
**32470486**	Ruxolitinib in treatment of severe coronavirus disease 2019 (COVID-19): A multicenter, single-blind, randomized controlled trial	Cao Y, et al.	J Allergy Clin Immunol	10.1016/j.jaci.2020.05.019	10.79	China	41	Pharmacological intervention	two-armed	50.00
**32492084**	Effect of Convalescent Plasma Therapy on Time to Clinical Improvement in Patients With Severe and Life-threatening COVID-19: A Randomized Clinical Trial	Li L, et al.	JAMA	10.1001/jama.2020.10044	56.27	China	103	Pharmacological intervention	two-armed	71.43
**32543164**	The comparison of the effectiveness of lincocin® and azitro® in the treatment of covid-19-associated pneumonia: A prospective study	Guvenmez O, et al.	J Popul Ther Clin Pharmacol	10.15586/jptcp.v27iSP1.684	NA	Turkey	24	Pharmacological intervention	two-armed	16.67
**32579195**	Effect of Colchicine vs Standard Care on Cardiac and Inflammatory Biomarkers and Clinical Outcomes in Patients Hospitalized With	Deftereos SG, et al.	JAMA network open	10.1001/jamanetworkopen.2020.13136	8.48	Greece	105	Pharmacological intervention	two-armed	65.71

	Coronavirus Disease 2019: The GRECCO-19 Randomized Clinical Trial									
**32603531**	Febuxostat therapy in outpatients with suspected COVID-19: A clinical trial	Davoodi L, et al.	International Journal of clinical practice	10.1111/ijcp.13600	NA	Iran	60	Pharmacological intervention	two-armed	36.11
**32661006**	A Randomized Clinical Trial of the Efficacy and Safety of Interferon β-1a in Treatment of Severe COVID-19	Davoudi-Monfared E, et al.	Antimicrobial Agents and Chemotherapy	10.1128/AAC.01061-20	NA	Iran	92	Pharmacological intervention	two-armed	57.14
**32671131**	Antiviral Activity and Safety of Darunavir/Cobicistat for the Treatment of COVID-19	Chen J, et al.	Open Forum Inf Dis	10.1093/ofid/ofaa241	3.84	China	30	Pharmacological intervention	two-armed	40.00
**32673060**	Hydroxychloroquine in Nonhospitalized Adults With Early COVID-19: A Randomized Trial	Skipper CP, et al.	Annals of Internal Medicine	10.7326/M20-4207	25.39	USA	491	Pharmacological intervention	two-armed	77.78
**32696396**	A Small-Scale Medication of Leflunomide as a Treatment of COVID-19 in an Open-Label Blank-Controlled Clinical Trial	Hu K, et al.	Virologica Sinica	10.1007/s12250-020-00258-7	4.33	China	10	Pharmacological intervention	two-armed	37.14
**32785710**	Methylprednisolone as Adjunctive Therapy for Patients Hospitalized With COVID-19 (Metcovid): A Randomised, Double-Blind, Phase IIb, Placebo-Controlled Trial	Jeronimo CMP, et al.	Clin Inf Dis	10.1093/cid/ciaa1177	9.08	Brazil	416	Pharmacological intervention	two-armed	70.27
**32811531**	Treatment of severe COVID-19 with human umbilical cord mesenchymal stem cells	Shu L, et al.	Stem Cell Res Ther	10.1186/s13287-020-01875-5	6.83	China	41	Pharmacological intervention	two-armed	37.14

**32812025**	Evaluation of the efficacy of sofosbuvir plus daclatasvir in combination with ribavirin for hospitalized COVID-19 patients with moderate disease compared with standard care: a single-centre, randomized controlled trial	Abbaspour Kasgari H, et al.	J Antimicrob Chemothr	10.1093/jac/dkaa332	5.79	Iran	48	Pharmacological intervention	two-armed	48.57
**32812039**	Sofosbuvir and daclatasvir compared with standard of care in the treatment of patients admitted to hospital with moderate or severe coronavirus infection (COVID-19): a randomized controlled trial	Sadeghi A, et al.	J Antimicrob Chemothr	10.1093/jac/dkaa334	5.79	Iran	66	Pharmacological intervention	two-armed	55.56
**32828135**	Hydroxychloroquine in the Treatment of COVID-19: A Multicenter Randomized Controlled Study	Abd-Elsalam S, et al.	Am J Trop Med Hyg	10.4269/ajtmh.20-0873	2.35	Egypt	175	Pharmacological intervention	two-armed	36.11
**32837847**	A Randomized, Open-label, Controlled Clinical Trial of Azvudine Tablets in the Treatment of Mild and Common COVID-19, A Pilot Study	Ren Z, et al.	Advanced Science (Weinheim)	10.1002/advs.202001435	16.81	China	20	Pharmacological intervention	two-armed	50.00
**32853672**	Safety and effectiveness of azithromycin in patients with COVID-19: An open-label randomised trial	Sekhavati E, et al.	Int J Antimicr Agents	10.1016/j.ijantimicag.2020.106143	5.28	Iran	111	Pharmacological intervention	two-armed	36.11
**32862111**	Interferon β-1b in treatment of severe COVID-19: A randomized clinical trial	Rahmani H, et al.	Int Immunopharmacol	10.1016/j.intimp.2020.106903	4.93	Iran	80	Pharmacological intervention	two-armed	51.43

**32871238**	Effect of calcifediol treatment and best available therapy versus best available therapy on intensive care unit admission and mortality among patients hospitalized for COVID-19: A pilot randomized clinical study	Entrenas Castillo M, et al.	J Steroid Biochem Mol Biol	10.1016/j.jsbmb.2020.105751	4.29	Spain	76	Pharmacological intervention	two-armed	38.89
**32876689**	Effect of Hydrocortisone on 21-Day Mortality or Respiratory Support Among Critically Ill Patients With COVID-19: A Randomized Clinical Trial	Dequin PF, et al.	JAMA	10.1001/jama.2020.16761	56.27	France	149	Pharmacological intervention	two-armed	67.57
**32876695**	Effect of Dexamethasone on Days Alive and Ventilator-Free in Patients With Moderate or Severe Acute Respiratory Distress Syndrome and COVID-19: The CoDEX Randomized Clinical Trial	Tomazini BM, et al.	JAMA	10.1001/jama.2020.17021	56.27	Brazil	299	Pharmacological intervention	two-armed	77.14
**32881359**	Bromhexine Hydrochloride Tablets for the Treatment of Moderate COVID-19: An Open-label Randomized Controlled Pilot Study	Li T, et al.	Clin Trans Sci	10.1111/cts.12881	4.69	China	18	Pharmacological intervention	two-armed	45.71
**32896292**	Azithromycin in addition to standard of care versus standard of care alone in the treatment of patients admitted to the hospital with severe COVID-19 in Brazil	Furtado RHM, et al.	The Lancet	10.1016/S0140-6736(20)31862-6	79.32	Brazil	447	Pharmacological intervention	two-armed	80.00

	(COALITION II): a randomised clinical trial									
**32910179**	Effect of Recombinant Human Granulocyte Colony-Stimulating Factor for Patients With Coronavirus Disease 2019 (COVID-19) and Lymphopenia: A Randomized Clinical Trial	Cheng LL, et al.	JAMA Internal Medicine	10.1001/jamainternmed.2020.5503	21.87	China	200	Pharmacological intervention	two-armed	68.57
**32923016**	Efficacy and safety of triazavirin therapy for coronavirus disease 2019: A pilot randomized controlled trial	Wu X, et al.	Engineering (Beijing)	10.1016/j.eng.2020.08.011	NA	China	42	Pharmacological intervention	two-armed	55.56
**32943404**	Intravenous methylprednisolone pulse as a treatment for hospitalised severe COVID-19 patients: results from a randomised controlled clinical trial	Edalatifard M, et al.	Eur Resp J	10.1183/13993003.02808-2020	16.67	Iran	68	Pharmacological intervention	two-armed	48.57
**32955081**	Treatment of COVID-19 Patients with Prolonged Post-Symptomatic Viral Shedding with Leflunomide -- a Single-Center, Randomized, Controlled Clinical Trial	Wang M, et al.	Clin Inf Dis	10.1093/cid/ciaa1417	9.08	China	50	Pharmacological intervention	two-armed	48.57
**32958718**	A prospective, randomized, open-label trial of early versus late favipiravir in hospitalized patients with COVID-19	Doi Y, et al.	Antimicrobial Agents and Chemotherapy	10.1128/AAC.01897-20	NA	Japan	89	Pharmacological intervention	two-armed	62.86
**32964918**	Double-blind, randomized, placebo-controlled trial with N-acetylcysteine for treatment of severe acute respiratory	de Alencar JCG, et al.	Clin Inf Dis	10.1093/cid/ciaa1443	9.08	Brazil	140	Pharmacological intervention	two-armed	45.95

	syndrome caused by COVID-19									
**32977137**	Therapeutic versus prophylactic anticoagulation for severe COVID-19: A randomized phase II clinical trial (HESACOVID)	Lemos ACB, et al.	Thromb Res	10.1016/j.thromres.2020.09.026	3.94	Brazil	20	Pharmacological intervention	two-armed	57.14
**32983936**	Effect of bromhexine on clinical outcomes and mortality in COVID-19 patients: A randomized clinical trial	Ansarin K, et al.	Bioimpacts	10.34172/bi.2020.27	3.83	Iran	78	Pharmacological intervention	two-armed	36.11
**32984784**	An open-label, randomized trial of the combination of IFN-κ plus TFF2 with standard care in the treatment of patients with moderate COVID-19	Fu W, et al.	EClinicalMedicine	10.1016/j.eclinm.2020.100547	NA	China	80	Pharmacological intervention	two-armed	62.86
**33134417**	Treating COVID-19 With Hydroxychloroquine (TEACH): A Multicenter, Double-Blind Randomized Controlled Trial in Hospitalized Patients	Ulrich RJ, et al.	Open Forum Inf Dis	10.1093/ofid/ofaa446	3.84	USA	128	Pharmacological intervention	two-armed	51.35
**33015643**	Anti-C5a antibody IFX-1 (vilobelimab) treatment versus best supportive care for patients with severe COVID-19 (PANAMO): an exploratory, open-label, phase 2 randomised controlled trial	Vlaar APJ, et al.	Lancet Reumatol	10.1016/S2665-9913(20)30341-6	8.14	The Netherlands	30	Pharmacological intervention	two-armed	62.86
**33031764**	Lopinavir-ritonavir in patients admitted to hospital with COVID-19 (RECOVERY): a randomised, controlled,	RECOVERY Collaborative Group.	The Lancet	10.1016/S0140-6736(20)32013-4	79.32	Great-Britain	5040	Pharmacological intervention	two-armed	69.44

	open-label, platform trial									
**33031652**	Effect of Hydroxychloroquine in Hospitalized Patients with Covid-19	RECOVERY Collaborative Group, et al.	NEJM	10.1056/NEJMoa2022926	91.25	Great-Britain	4716	Pharmacological intervention	two-armed	61.11
**33080005**	Effect of Tocilizumab vs Standard Care on Clinical Worsening in Patients Hospitalized With COVID-19 Pneumonia: A Randomized Clinical Trial	Salvarani C, et al.	JAMA Internal Medicine	10.1001/jamainternmed.2020.6615	21.87	Italy	126	Pharmacological intervention	two-armed	66.67
**33080017**	Effect of Tocilizumab vs Usual Care in Adults Hospitalized With COVID-19 and Moderate or Severe Pneumonia: A Randomized Clinical Trial	Hermine O, et al.	JAMA Internal Medicine	10.1001/jamainternmed.2020.6820	21.87	France	131	Pharmacological intervention	two-armed	86.11
**33082342**	A pragmatic randomized controlled trial reports lack of efficacy of hydroxychloroquine on coronavirus disease 2019 viral kinetics	Lyngbakken MN, et al.	Nature Communications	10.1038/s41467-020-19056-6	14.92	Norway	53	Pharmacological intervention	two-armed	54.29
**33085857**	Efficacy of Tocilizumab in Patients Hospitalized with Covid-19	Stone JH, et al.	NEJM	10.1056/NEJMoa2028836	91.25	USA	243	Pharmacological intervention	two-armed	56.76
**33087047**	The use of intravenous immunoglobulin gamma for the treatment of severe coronavirus disease 2019: a randomized placebo-controlled double-blind clinical trial	Gharebaghi N, et al.	BMC Infectious Diseases	10.1186/s12879-020-05507-4	3.09	Iran	59	Pharmacological intervention	two-armed	37.84
**33093056**	Convalescent plasma in the management of moderate covid-19 in adults in India: open	Agarwal A, et al.	BMJ	10.1136/bmj.m3939	39.89	India	464	Pharmacological intervention	two-armed	80.56

	label phase II multicentre randomised controlled trial (PLACID Trial)									
**33165621**	Effect of Hydroxychloroquine on Clinical Status at 14 Days in Hospitalized Patients With COVID-19: A Randomized Clinical Trial	Self WH, et al.	JAMA	10.1001/jama.2020.22240	56.27	USA	479	Pharmacological intervention	two-armed	75.00
**33111980**	Ozone as adjuvant support in the treatment of COVID-19: A preliminary report of probiozovid trial	Araimo F, et al.	J Med Virol	10.1002/jmv.26636	2.33	Italy	28	Pharmacological intervention	two-armed	33.33
**33166179**	Hydroxychloroquine vs. Azithromycin for Hospitalized Patients with COVID-19 (HAHPS): Results of a Randomized, Active Comparator Trial	Brown SM, et al.	Annals of the American Thoracic Society	10.1513/AnnalsATS.202008-940OC	6.83	USA	85	Pharmacological intervention	two-armed	54.29
**33181328**	Randomized Controlled Open Label Trial on the Use of Favipiravir Combined with Inhaled Interferon beta-1b in Hospitalized Patients with Moderate to Severe COVID-19 Pneumonia	Khamis F, et al.	Int J Infect Dis	10.1016/j.ijid.2020.11.008	3.62	Oman	89	Pharmacological intervention	two-armed	34.29
**33184146**	Short term, high-dose vitamin D supplementation for COVID-19 disease: a randomised, placebo-controlled, study (SHADE study)	Rastogi A, et al.	BMJ	10.1136/postgradmedj-2020-139065	39.89	India	40	Pharmacological intervention	two-armed	43.24
**33189161**	Safety and efficacy of inhaled nebulised interferon beta-1a (SNG001) for treatment of SARS-CoV-2 infection: a	Monk PD, et al.	Lancet Respir Med	10.1016/S2213-2600(20)30511-7	30.70	Great-Britain	101	Pharmacological intervention	two-armed	66.67

	randomised, double-blind, placebo-controlled, phase 2 trial									
**33214093**	Evaluating the effects of Intravenous Immunoglobulin (IVIg) on the management of severe COVID-19 cases: A randomized controlled trial	Tabarsi P, et al.	Int Immunopharmacol	10.1016/j.intimp.2020.107205	4.93	Iran	84	Pharmacological intervention	two-armed	38.89
**33212256**	Efficacy and Safety of Favipiravir, an Oral RNA-Dependent RNA Polymerase Inhibitor, in Mild-to-Moderate COVID-19: A Randomized, Comparative, Open-Label, Multicenter, Phase 3 Clinical Trial	Udwadia ZF, et al.	Int J Infect Dis	10.1016/j.ijid.2020.11.142	3.62	India	150	Pharmacological intervention	two-armed	71.43
**33204764**	Safety of Hydroxychloroquine Among Outpatient Clinical Trial Participants for COVID-19	Lofgren SM, et al.	Open Forum Inf Dis	10.1093/ofid/ofaa500	3.84	USA	491	Pharmacological intervention	two-armed	21.62
**33225306**	Intravenous Immunoglobulin Plus Methylprednisolone Mitigate Respiratory Morbidity in Coronavirus Disease 2019	Sakoulas G, et al.	Crit Care Explor	10.1097/CCE.0000000000000280	NA	USA	34	Pharmacological intervention	two-armed	55.56
**33232588**	A Randomized Trial of Convalescent Plasma in Covid-19 Severe Pneumonia	Simonovich VA, et al.	NEJM	10.1056/NEJMoa2031304	91.25	Argentina	334	Pharmacological intervention	two-armed	69.44
**33247380**	Do Zinc Supplements Enhance the Clinical Efficacy of Hydroxychloroquine?: a Randomized, Multicenter Trial	Abd-Elsalam S, et al.	Biological trace element research	10.1007/s12011-020-02512-1	3.74	Egypt	191	Pharmacological intervention	two-armed	36.11

**33278747**	Pentoxifylline decreases serum LDH levels and increases lymphocyte count in COVID-19 patients: Results from an external pilot study	Maldonado V, et al.	Int Immunopharmacol	10.1016/j.intimp.2020.107209	4.93	Mexico	54	Pharmacological intervention	two-armed	30.56
**33409026**	The Role of Vitamin C as Adjuvant Therapy in COVID-19	Kumari P, et al.	Cureus	10.7759/cureus.11779	NA	Pakistan	150	Pharmacological intervention	two-armed	13.89
**33264337**	A multicenter, randomized, open-label, controlled trial to evaluate the efficacy and tolerability of hydroxychloroquine and a retrospective study in adult patients with mild to moderate coronavirus disease 2019 (COVID-19)	Chen CP, et al.	Plos One	10.1371/journal.pone.0242763	3.24	Taiwan	33	Pharmacological intervention	two-armed	45.71
**33306283**	Baricitinib plus Remdesivir for Hospitalized Adults with Covid-19	Kalil AC, et al.	NEJM	10.1056/NEJMoa2031994	91.25	USA	1033	Pharmacological intervention	two-armed	50.00
**33317461**	Effect of Arbidol (Umifenovir) on COVID-19: a randomized controlled trial	Nojomi M, et al.	BMC Infectious Diseases	10.1186/s12879-020-05698-w	3.09	Iran	100	Pharmacological intervention	two-armed	42.86
**33332779**	Tocilizumab in Patients Hospitalized with Covid-19 Pneumonia	Salama C, et al.	NEJM	10.1056/NEJMoa2030340	91.25	USA	388	Pharmacological intervention	two-armed	56.76
**33338232**	Sofosbuvir and daclatasvir for the treatment of COVID-19 outpatients: a double-blind, randomized controlled trial	Roozbeh F, et al.	J Antimicr Chemother	10.1093/jac/dkaa501	5.79	Iran	55	Pharmacological intervention	two-armed	33.33
**33356051**	A Neutralizing Monoclonal Antibody for Hospitalized Patients with Covid-19	ACTIV-3/TICO LY-CoV555 Study	NEJM	10.1056/NEJMoa2033130	91.25	Denmark	326	Pharmacological intervention	two-armed	56.76

		Group, Lundgren JD, et al.								
**33356977**	Awake-Prone Positioning Strategy for Non-Intubated Hypoxic Patients with COVID-19: A Pilot Trial with Embedded Implementation Evaluation	Taylor SP, et al.	Annals of the American Thoracic Society	10.1513/AnnalsATS.202009-1164OC	6.83	USA	41	Non-Pharmacological intervention	two-armed	45.71
**33361100**	Early use of nitazoxanide in mild Covid-19 disease: randomised, placebo-controlled trial	Rocco PRM, et al.	Eur Respir J	10.1183/13993003.03725-2020	16.67	Brazil	475	Pharmacological intervention	two-armed	59.46
**33421928**	Safety and efficacy of ozone therapy in mild to moderate COVID-19 patients: A phase 1/11 randomized control trial (SEOT study)	Shah M, et al.	Int Immunopharmacol	10.1016/j.intimp.2020.107301	4.93	India	60	Pharmacological intervention	two-armed	31.43
**33068293**	The value of high-flow nasal cannula oxygen therapy in treating novel coronavirus pneumonia	Teng XB, et al.	Eur J Clin Invest	10.1111/eci.13435	4.69	China	12	Non-Pharmacological intervention	two-armed	14.29
**33615173**	Treatment with an Anti-CK2 Synthetic Peptide Improves Clinical Response in COVID-19 Patients with Pneumonia. A Randomized and Controlled Clinical Trial	Cruz LR, et al.	ACS Pharmacol Transl Sci	10.1021/acsptsci.0c00175	NA	Cuba	20	Pharmacological intervention	two-armed	30.56
**33525212**	Efficacy and safety of sofosbuvir/ ledipasvir in treatment of patients with COVID-19; A randomized clinical trial	Khalili H, et al.	Acta Biomed	10.23750/abm.v91i4.10877	NA	Iran	90	Pharmacological intervention	two-armed	47.22
**33406353**	Early High-Titer Plasma Therapy to Prevent Severe Covid-19 in Older Adults	Libster R, et al.	NEJM	10.1056/NEJMoa2033700	91.25	Argentina	160	Pharmacological intervention	two-armed	64.86

**33486496**	Early Use of Corticosteroid May Prolong SARS-CoV-2 Shedding in Non-Intensive Care Unit Patients with COVID-19 Pneumonia: A Multicenter, Single-Blind, Randomized Control Trial	Tang X, et al.	Respiration	10.1159/000512063	3.58	China	86	Pharmacological intervention	two-armed	58.33
**33575961**	Results and Prospects of Using Activator of Hematopoietic Stem Cell Differentiation in Complex Therapy for Patients with COVID-19	Khavinson VK, et al.	Stem Cell Rev Rep	10.1007/s12015-020-10087-6	5.74	Russia	92	Pharmacological intervention	two-armed	8.57
**33542047**	Beneficial effects of colchicine for moderate to severe COVID-19: a randomised, double-blinded, placebo-controlled clinical trial	Lopes MI, et al.	RMD open	10.1136/rmdopen-2020-001455	5.12	Brazil	75	Pharmacological intervention	two-armed	41.67
**33568628**	Effect of human umbilical cord-derived mesenchymal stem cells on lung damage in severe COVID-19 patients: a randomized, double-blind, placebo-controlled phase 2 trial	Shi L, et al.	Signal Transduct Target Ther	10.1038/s41392-021-00488-5	18.19	China	101	Pharmacological intervention	two-armed	63.89
**33493450**	Effect of anakinra versus usual care in adults in hospital with COVID-19 and mild-to-moderate pneumonia (CORIMUNO-ANA-1): a randomised controlled trial	CORIMUNO-19 Collaborative group.	Lancet Respir Med	10.1016/S2213-2600(20)30556-7	30.70	France	116	Pharmacological intervention	two-armed	80.56
**33464336**	Effect of Discontinuing vs Continuing Angiotensin-Converting Enzyme Inhibitors and Angiotensin II Receptor Blockers on Days Alive and Out of the Hospital	Lopes RD, et al.	JAMA	10.1001/jama.2020.25864	56.27	USA	740	Pharmacological intervention	two-armed	75.00

	in Patients Admitted With COVID-19: A Randomized Clinical Trial									
**33422263**	Continuation versus discontinuation of renin-angiotensin system inhibitors in patients admitted to hospital with COVID-19: a prospective, randomised, open-label trial	Cohen JB, et al.	Lancet Respir Med	10.1016/S2213-2600(20)30558-0	30.70	USA	152	Pharmacological intervention	two-armed	74.29
**33573699**	Safety and effectiveness of high-dose vitamin C in patients with COVID-19: a randomized open-label clinical trial	JamaliMoghadamSiahkali S, et al.	Eur J Med Res	10.1186/s40001-021-00490-1	2.18	Iran	60	Pharmacological intervention	two-armed	41.67
**33472855**	Effect of tocilizumab on clinical outcomes at 15 days in patients with severe or critical coronavirus disease 2019: randomised controlled trial	Veiga VC, et al.	BMJ	10.1136/bmj.n84	39.89	Brazil	129	Pharmacological intervention	two-armed	80.56
**33595634**	Effect of a Single High Dose of Vitamin D3 on Hospital Length of Stay in Patients With Moderate to Severe COVID-19: A Randomized Clinical Trial	Murai IH, et al.	JAMA	10.1001/jama.2020.26848	56.27	Brazil	240	Pharmacological intervention	two-armed	69.44
**33633920**	Proxalutamide Significantly Accelerates Viral Clearance and Reduces Time to Clinical Remission in Patients with Mild to Moderate COVID-19: Results from a Randomized, Double-Blinded, Placebo-Controlled Trial	Cadegiani FA, et al.	Cureus	10.7759/cureus.13492	NA	USA	236	Pharmacological intervention	two-armed	13.51

**33643746**	Early Antiandrogen Therapy With Dutasteride Reduces Viral Shedding, Inflammatory Responses, and Time-to-Remission in Males With COVID-19: A Randomized, Double-Blind, Placebo-Controlled Interventional Trial (EAT-DUTA AndroCoV Trial - Biochemical)	Cadegiani FA, et al.	Cureus	10.7759/cureus.13047	NA	Brazil	130	Pharmacological intervention	two-armed	32.43
**33420963**	Pilot trial of high-dose vitamin C in critically ill COVID-19 patients	Zhang J, et al.	Ann Intensive Care	10.1186/s13613-020-00792-3	6.93	China	56	Pharmacological intervention	two-armed	63.89
**33556319**	Peginterferon lambda for the treatment of outpatients with COVID-19: a phase 2, placebo-controlled randomised trial	Feld JJ, et al.	Lancet Respir Med	10.1016/S2213-2600(20)30566-X	30.70	Canada	60	Pharmacological intervention	two-armed	70.27
**33585890**	A Phase II Safety and Efficacy Study on Prognosis of Moderate Pneumonia in COVID-19 patients with Regular Intravenous Immunoglobulin Therapy	Raman RS, et al.	J Infec Dis	10.1093/infdis/jiab098	5.23	India	100	Pharmacological intervention	two-armed	37.14
**33473017**	Chloroquine nasal drops in asymptomatic & mild COVID-19: An exploratory randomized clinical trial	Thakar A, et al.	Indian J Med Res	10.4103/ijmr.IJMR_3665_20	2.38	India	60	Pharmacological intervention	two-armed	41.67
**33534047**	Methylprednisolone in adults hospitalized with COVID-19 pneumonia : An open-label randomized trial (GLUCOCOVID)	Corral-Gudino L, et al.	Wien Klin Wochenschr	10.1007/s00508-020-01805-8	1.70	Spain	64	Pharmacological intervention	two-armed	58.33

**33621601**	Progesterone in Addition to Standard of Care vs Standard of Care Alone in the Treatment of Men Hospitalized With Moderate to Severe COVID-19: A Randomized, Controlled Pilot Trial	Ghandehari S, et al.	Chest	10.1016/j.chest.2021.02.024	9.41	USA	42	Pharmacological intervention	two-armed	69.44
**33631066**	Tocilizumab in Hospitalized Patients with Severe Covid-19 Pneumonia	Rosas IO, et al.	NEJM	10.1056/NEJMoa2028700	91.25	Great-Britain	452	Pharmacological intervention	two-armed	63.89
**33400390**	Umbilical cord mesenchymal stem cells for COVID-19 acute respiratory distress syndrome: A double-blind, phase 1/2a, randomized controlled trial	Lanzoni G, et al.	Stem cells transl med	10.1002/sctm.20-0472	6.94	USA	28	Pharmacological intervention	two-armed	48.65
**33545096**	Azithromycin in patients admitted to hospital with COVID-19 (RECOVERY): a randomised, controlled, open-label, platform trial	RECOVERY Collaborative Group.	Lancet	10.1016/S0140-6736(21)00149-5	79.32	Great-Britain	7763	Pharmacological intervention	two-armed	75.00
**32876697**	Effect of Hydrocortisone on Mortality and Organ Support in Patients With Severe COVID-19: The REMAP-CAP COVID-19 Corticosteroid Domain Randomized Clinical Trial	Angus DC, et al.	JAMA	10.1001/jama.2020.17022	56.27	USA	403	Pharmacological intervention	>2 arms	68.57
**33115675**	Clinical Outcomes and Plasma Concentrations of Baloxavir Marboxil and Favipiravir in COVID-19 Patients: An Exploratory	Lou Y, et al.	Eur J Pharm Sci	10.1016/j.ejps.2020.105631	4.38	China	30	Pharmacological intervention	>2 arms	36.11

	Randomized, Controlled Trial									
**33113295**	SARS-CoV-2 Neutralizing Antibody LY-CoV555 in Outpatients with Covid-19	Chen P, et al.	NEJM	10.1056/NEJMoa2029849	91.25	USA	467	Pharmacological intervention	>2 arms	44.44
**33378989**	Tocilizumab combined with favipiravir in the treatment of COVID-19: A multicenter trial in a small sample size	Zhao H, et al.	Biomed Pharmacother	10.1016/j.biopha.2020.110825	6.53	China	14	Pharmacological intervention	>2 arms	28.57
**33251500**	Randomized double-blinded placebo-controlled trial of hydroxychloroquine with or without azithromycin for virologic cure of non-severe Covid-19	Omrani AS, et al.	EClinicalMedicine	10.1016/j.eclinm.2020.100645	NA	Qatar	456	Pharmacological intervention	>2 arms	67.57
**33332778**	REGN-COV2, a Neutralizing Antibody Cocktail, in Outpatients with Covid-19	Weinreich DM, et al.	NEJM	10.1056/NEJMoa2035002	91.25	USA	275	Pharmacological intervention	>2 arms	54.05
**32706953**	Hydroxychloroquine with or without Azithromycin in Mild-to-Moderate Covid-19	Cavalcanti AB, et al.	NEJM	10.1056/NEJMoa2019014	91.25	Brazil	665	Pharmacological intervention	>2 arms	71.43
**32758689**	SARS-CoV-2 clearance in COVID-19 patients with Novaferon treatment: A randomized, open-label, parallel-group trial	Zheng F, et al.	Int J Inf Dis	10.1016/j.ijid.2020.07.053	3.62	China	89	Pharmacological intervention	>2 arns	41.67
**32765274**	No Statistically Apparent Difference in Antiviral Effectiveness Observed Among Ribavirin Plus Interferon-Alpha, Lopinavir/Ritonavir Plus Interferon-Alpha, and Ribavirin Plus Lopinavir/Ritonavir Plus Interferon-Alpha in	Huang YQ, et al.	Front Pharmacol	10.3389/fphar.2020.01071	5.81	China	101	Pharmacological intervention	>2 arms	54.29

	Patients With Mild to Moderate Coronavirus Disease 2019: Results of a Randomized, Open-Labeled Prospective Study									
**32821939**	Effect of Remdesivir vs Standard Care on Clinical Status at 11 Days in Patients With Moderate COVID-19: A Randomized Clinical Trial	Spinner CD, et al.	JAMA	10.1001/jama.2020.16349	56.27	USA	596	Pharmacological intervention	>2 arms	74.29
**32838353**	Efficacy and Safety of Lopinavir/Ritonavir or Arbidol in Adult Patients with Mild/Moderate COVID-19: An Exploratory Randomized Controlled Trial	Li Y, et al.	Med (NY)	10.1016/j.medj.2020.04.001	NA	China	86	Pharmacological intervention	>2 arns	44.44
**33475701**	Effect of Bamlanivimab as Monotherapy or in Combination With Etesevimab on Viral Load in Patients With Mild to Moderate COVID-19: A Randomized Clinical Trial	Gottlieb RL, et al.	JAMA	10.1001/jama.2021.0202	56.27	USA	592	Pharmacological intervention	>2 arms	61.11
**33576820**	Effect of High-Dose Zinc and Ascorbic Acid Supplementation vs Usual Care on Symptom Length and Reduction Among Ambulatory Patients With SARS-CoV-2 Infection: The COVID A to Z Randomized Clinical Trial	Thomas S, et al.	JAMA Netw Open	10.1001/jamanetworkopen.2021.0369	8.48	USA	214	Pharmacological intervention	>2 arms	65.71
**33681731**	Hydroxychloroquine with or without azithromycin for treatment of early SARS-CoV-2 infection	Johnston C, et al.	EClinicalMedicine	10.1016/j.eclinm.2021.100773	NA	USA	231	Pharmacological intervention	>2 arms	59.46

	among high-risk outpatient adults: A randomized clinical trial									
**33599247**	Ivermectin shows clinical benefits in mild to moderate COVID19: A randomised controlled double-blind, dose-response study in Lagos	Babalola OE, et al.	QJM	10.1093/qjmed/hcab035	3.21	Nigeria	63	Pharmacological intervention	>2 arms	36.11
**33628506**	A Randomized, Double-Blind, Multicenter Clinical Study Comparing the Efficacy and Safety of a Drug Combination of Lopinavir/Ritonavir-Azithromycin, Lopinavir/Ritonavir-Doxycycline, and Azithromycin-Hydroxychloroquine for Patients Diagnosed with Mild to Moderate COVID-19 Infections	Purwati, et al.	Biochemistry Research International	10.1155/2021/6685921	NA	Indonesia	754	Pharmacological intervention	>2 arms	30.56
**33631065**	Interleukin-6 Receptor Antagonists in Critically Ill Patients with Covid-19	REMAP-CAP Investigators, et al.	NEJM	10.1056/NEJMoa2100433	91.25	Great-Britain	895	Pharmacological intervention	>2 arms	58.33
**32678530**	Dexamethasone in Hospitalized Patients with Covid-19	RECOVERY Collaborative Group, et al.	NEJM	10.1056/NEJMoa2021436	91.25	Great-Britain	6425	Pharmacological intervention	two-armed	58.33
**33718487**	Self-proning in COVID-19 patients on low-flow oxygen therapy: a cluster randomised controlled trial	Kharat A, et al.	ERJ Open Res	10.1183/23120541.00692-2020	NA	Switzer-land	27	Non-Pharmacological intervention	two-armed	57.14
**32674126**	Hydroxychloroquine for Early Treatment of Adults with Mild Covid-19: A Randomized-Controlled Trial	Mitjà O, et al.	Clinical Infectious Diseases	10.1093/cid/ciaa1009	9.08	Spain	353	Pharmacological intervention	two-armed	54.29
**32676976**	Exploring an Integrative Therapy for Treating COVID-19: A	Wang JB, et al.	Chin J Integr Med	10.1007/s11655-	1.98	China	48	Traditional Medicine	two-armed	44.44

	Randomized Controlled Trial			020-3426-7						
**32781283**	Efficacy of Huoxiang Zhengqi dropping pills and Lianhua Qingwen granules in treatment of COVID-19: A randomized controlled trial	Xiao M, et al.	Pharmacol Res	10.1016/j.phrs.2020.105126	7.66	China	283	Traditional Medicine	>2 arms	27.78
**32874913**	Efficacy of herbal medicine (Xuanfei Baidu decoction) combined with conventional drug in treating COVID-19:A pilot randomized clinical trial	Xiong WZ, et al.	Integr Med Res	10.1016/j.imr.2020.100489	2.37	China	42	Traditional Medicine	>2 arms	36.11
**32795330**	Auxora versus standard of care for the treatment of severe or critical COVID-19 pneumonia: results from a randomized controlled trial	Miller J, et al.	Crit Care	10.1186/s13054-020-03220-x	9.10	USA	30	Traditional Medicine	two-armed	38.89
**33519133**	An Open Clinical Evaluation Of Selected Siddha Regimen In Expediting The Management Of Covid-19 -A Randomized Controlled Study	Chitra SM, et al.	J Ayurveda Integr Med	10.1016/j.jaim.2021.01.002	NA	India	200	Traditional Medicine	two-armed	37.14
**33552315**	The preventive effect of Xuebijing injection against cytokine storm for severe patients with COVID-19: A prospective randomized controlled trial	Luo Z, et al.	Eur J Integr Med	10.1016/j.eujim.2021.101305	1.31	China	60	Traditional Medicine	two-armed	52.78
**33596494**	Randomized placebo-controlled pilot clinical trial on the efficacy of ayurvedic treatment regime on COVID-19 positive patients	Devpura G, et al.	Phytomedicine	10.1016/j.phymed.2021.153494	5.34	India	100	Traditional Medicine	two-armed	41.67

**33495752**	The effect of early treatment with ivermectin on viral load, symptoms and humoral response in patients with non-severe COVID-19: A pilot, double-blind, placebo-controlled, randomized clinical trial	Chaccour C, et al.	EClinicalMedicine	10.1016/j.eclinm.2020.100720	NA	Spain	24	Pharmacological intervention	two-armed	64.86
**33180097**	Fluvoxamine vs Placebo and Clinical Deterioration in Outpatients With Symptomatic COVID-19: A Randomized Clinical Trial	Lenze EJ, et al.	JAMA	10.1001/jama.2020.22760	56.27	USA	181	Pharmacological intervention	two-armed	62.16
**33607104**	No clinical benefit of high dose corticosteroid administration in patients with COVID-19: A preliminary report of a randomized clinical trial	Jamaati H, et al.	Eur J Pharmacol	10.1016/j.ejphar.2021.173947	2.95	Iran	50	Pharmacological intervention	two-armed	30.56

*based on affiliation of corresponding author
