# Supplementary material for: Poor reporting quality of randomized controlled trials comparing treatments of COVID-19–A retrospective cross-sectional study on the first year of publications
Source: PLoS One. 2023 Oct 16;18(10):e0292860. doi: 10.1371/journal.pone.0292860 (PMC10578566; doi:10.1371/journal.pone.0292860)

Supplementary S3. Boxplots of investigated factors

1. **Percentage adherence and tertiles of impact factor (n=108)**

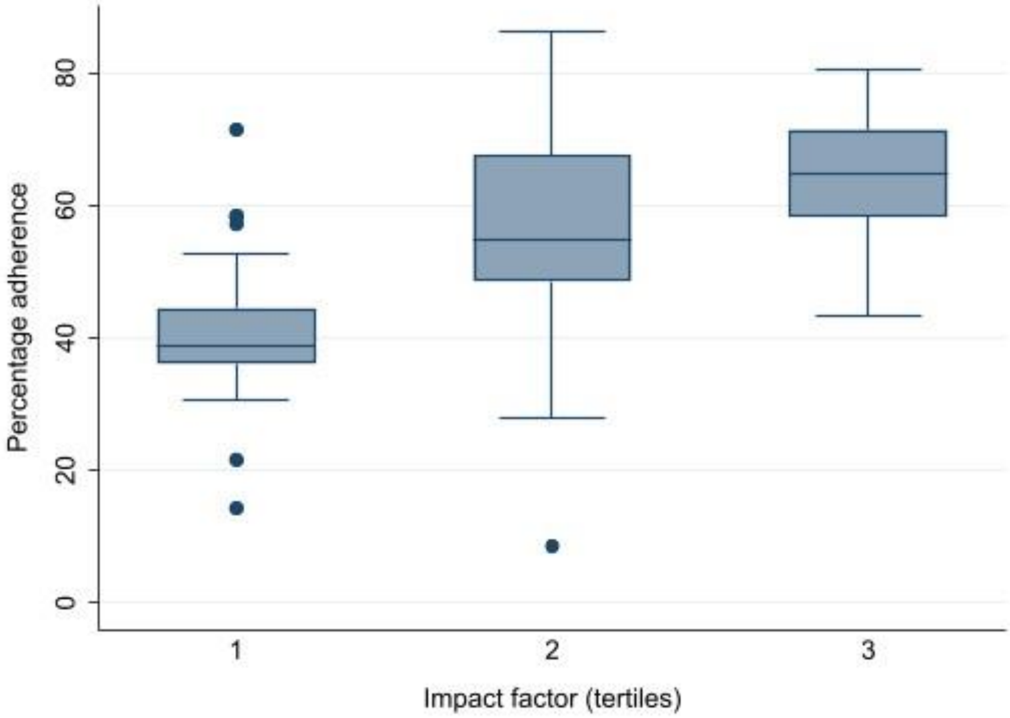

2. **Percentage adherence and tertiles of month of publication (n=127)**

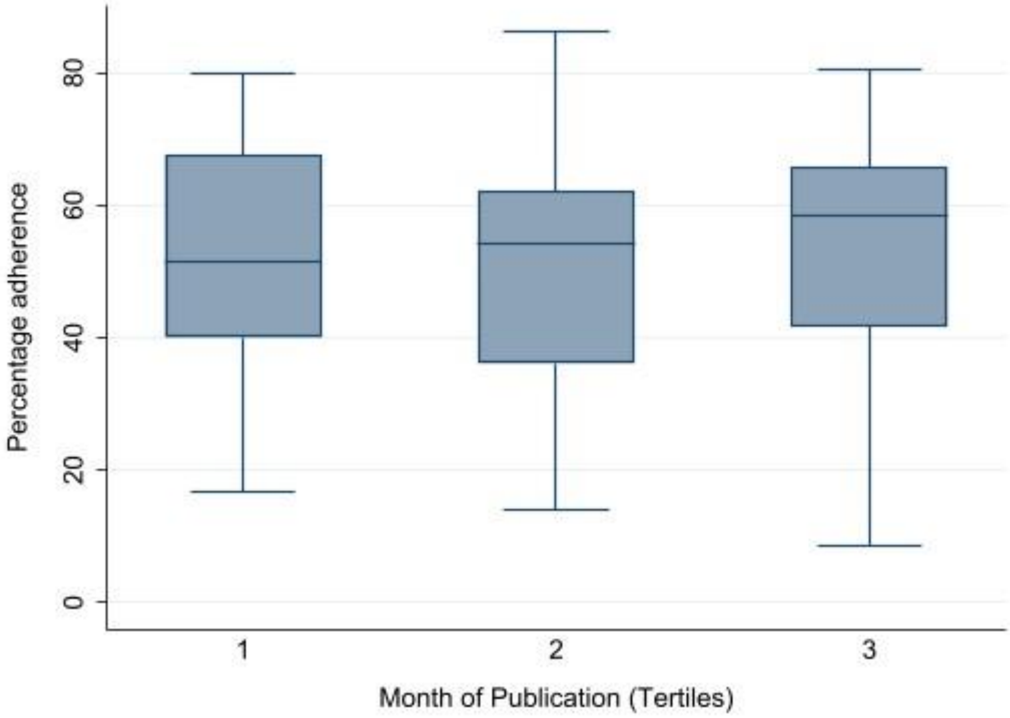

3. Percentage adherence and journal endorsement of CONSORT (n=127)

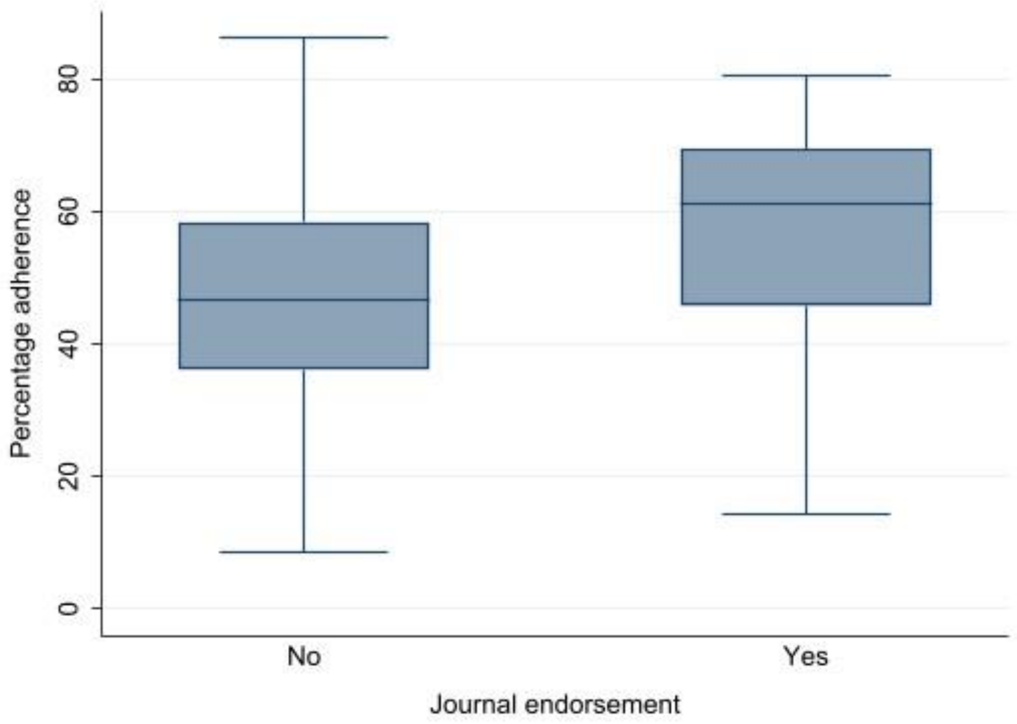

4. Percentage adherence and author referral to CONSORT (n=127)

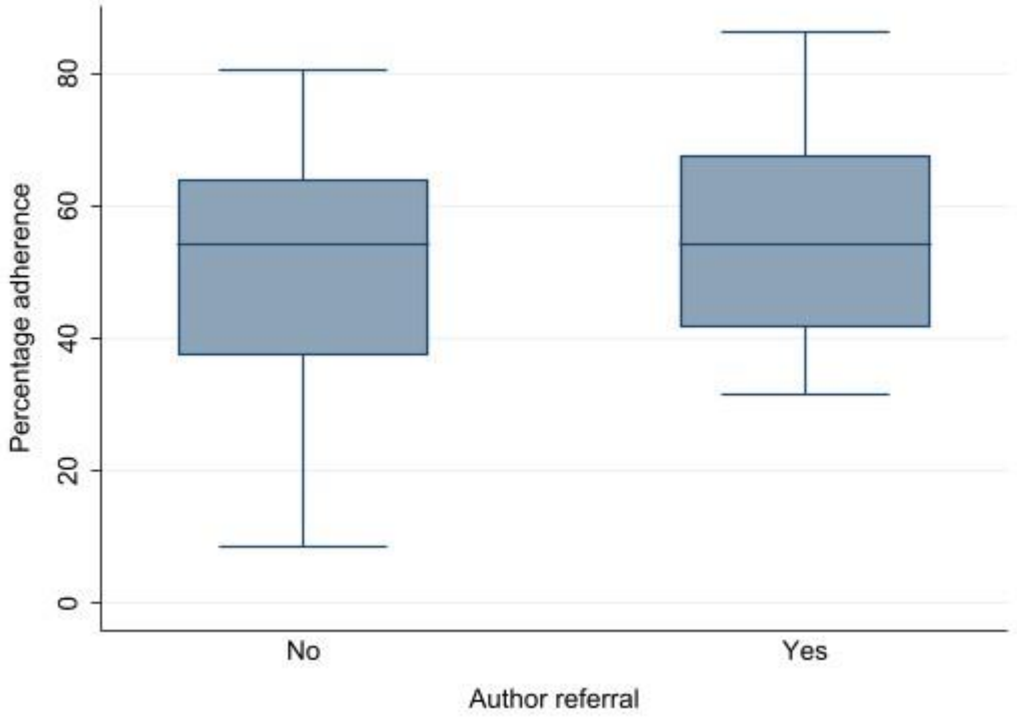

Supplement: S3 File — (PDF) [file pone.0292860.s003.pdf]
