# Supplementary material for: Poor reporting quality of randomized controlled trials comparing treatments of COVID-19–A retrospective cross-sectional study on the first year of publications
Source: PLoS One. 2023 Oct 16;18(10):e0292860. doi: 10.1371/journal.pone.0292860 (PMC10578566; doi:10.1371/journal.pone.0292860)
Supplement: S4 File — (PDF) [file pone.0292860.s004.pdf]

Supplementary S4. Linear regression models for separate predictors

Independent variable	Point estimate of change in percentage adherence			P-value
	β	95% CI	SE	
Country of origin[a]				
Brazil	6.23	-4.63 to 17.09	5.49	0.258
Great Britain	7.93	-5.32 to 21.19	6.69	0.238
China	-7.10	-15.50 to 1.31	4.25	0.097
India	-8.72	-21.30 to 3.87	6.36	0.173
Iran	-13.95	-24.04 to -3.86	5.10	0.007
Other countries	-6.70	-15.04 to 1.64	4.21	0.114
Intercept	56.75	50.65 to 62.85	3.08	<0.001

[a] Reference country: USA
R2=0.1396

Independent variable	Point estimate of change in percentage adherence			P-value
	β	95% CI	SE	
Journal endorsement CONSORT	11.33	5.85 to 16.81	2.77	<0.001
Intercept	46.94	43.14 to 50.73	1.92	<0.001

R2=0.1180

Independent variable	Point estimate of change in percentage adherence			P-value
	β	95% CI	SE	
Author referral CONSORT	2.44	-4.33 to 9.22	3.42	0.477
Intercept	51.78	48.43 to 55.13	1.70	<0.001

R2=0.0041

Independent variable	Point estimate of change in percentage adherence			P-value
	β	95% CI	SE	
Impact factor (continuously)	0.25	0.17 to 0.33	0.04	<0.001
Intercept	46.52	42.98 to 50.06	1.79	<0.001
Impact factor (tertiles*)				
2nd tertile	15.22	9.43 to 21.00	2.91	<0.001
3rd tertile	24.45	18.62 o 30.28	2.94	<0.001
Intercept	40.76	36.70 to 44.83	2.05	<0.001

* Reference category: first tertile
R2 when impact factor modelled continuously: 0.2586
R2 when impact factor modelled in tertiles: 0.4032

Independent variable		Point estimate of change in percentage adherence			P-value
		β	95% CI	SE	
Month of publication (continuously)		-0.22	-1.25 to 0.82	0.52	0.679
	Intercept	54.16	45.15 to 63.17	4.55	<0.001
Month of publication (tertiles*)					
	2nd tertile	-2.77	-9.64 to 4.09	3.47	0.426
	3rd tertile	0.82	-6.45 to 8.09	3.67	0.824
	Intercept	53.09	48.39 to 57.78	2.37	<0.001

* Reference category: first tertile
R2 when month of publication modelled continuously: 0.0014
R2 when month of publication modelled in tertiles: 0.0084
