# Supplementary material for: Poor reporting quality of randomized controlled trials comparing treatments of COVID-19–A retrospective cross-sectional study on the first year of publications
Source: PLoS One. 2023 Oct 16;18(10):e0292860. doi: 10.1371/journal.pone.0292860 (PMC10578566; doi:10.1371/journal.pone.0292860)
Supplement: S5 File — (PDF) [file pone.0292860.s005.pdf]

Supplementary S5. Exploratory multiple regression model with continuous variables

Independent variable	Point estimate of change in percentage adherence			P-value
	β	95% CI	SE	
Journal endorsement CONSORT	2.50	-4.23 to 9.22	3.39	0.463
Author referral CONSORT	9.50	2.90 to 16.09	3.32	0.005
Country of origin[a]				
Brazil	7.48	-2.34 to 17.30	4.95	0.134
Great Britain	0.15	-11.72 to 12.03	5.98	0.980
China	-4.45	-13.13 to 4.23	4.37	0.312
India	-6.90	-19.19 to 5.38	6.19	0.268
Iran	-11.49	-21.88 to -1.10	5.24	0.031
Other countries	-2.71	-10.99 to 5.58	4.18	0.518
Impact factor	0.20	0.09 to 0.32	0.06	0.001
Month of publication	-0.24	-1.30 to 0.81	0.53	0.648
Intercept	48.81	36.21 to 61.40	6.34	<0.001

[a] Reference country: USA

The table shows the associations of the prespecified independent variables (predictors) with the percentage adherence to the CONSORT checklist according to a multiple linear regression model. Overall regression model: Number of observation n=108 (19 publications published in journals without an available IF were excluded); R2 = 0.3876; adjusted R2 = 0.3245; F (10,97) = 6.14, p<0.001.
